# A Cytokine-Like Protein Dickkopf-Related Protein 3 Is Atheroprotective

**DOI:** 10.1161/CIRCULATIONAHA.117.027690

**Published:** 2017-09-11

**Authors:** Baoqi Yu, Stefan Kiechl, Dan Qi, Xiaocong Wang, Yanting Song, Siegfried Weger, Agnes Mayr, Alexandra Le Bras, Eirini Karamariti, Zhongyi Zhang, Ivan del Barco Barrantes, Christof Niehrs, Georg Schett, Yanhua Hu, Wen Wang, Johann Willeit, Aijuan Qu, Qingbo Xu

**Affiliations:** From Cardiovascular Division, King’s College London British Heart Foundation Centre, London, United Kingdom (B.Y., X.W., A.L.B., E.K., Z.Z., Y.H., Q.X.); Department of Neurology, Medical University of Innsbruck, Austria (S.K., J.W.); Department of Physiology and Pathophysiology, Capital Medical University, Beijing, China (D.Q., Y.S., A.Q.); Department of Internal and Laboratory Medicine, Bruneck Hospital, Italy (S.W., A.M.); Division of Molecular Embryology, German Cancer Research Center (DKFZ) Heidelberg Germany and Zentrum für Molekulare Biologie der Universität Heidelberg (ZMBH) Alliance, Heidelberg, Germany (I.d.B.B., C.N.); Institute of Molecular Biology, Mainz, Germany (C.N.); Department of Internal Medicine, Institute for Clinical Immunology, Friedrich-Alexander-University Erlangen-Nuremberg, Germany (G.S.); The Key Laboratory of Cardiovascular Remodelling and Function Research, Chinese Ministry of Education and Chinese Ministry of Health, Qilu Hospital, Shandong University, Jinan, China (Y.H., Q.X.); and Institute of Bioengineering, Queen Mary University of London, United Kingdom (W.W.)

**Keywords:** animal model, atherosclerosis, DKK3, endothelial cells, population study

## Abstract

**Background:**

Dickkopf-related protein 3 (DKK3) is a secreted protein that is involved in the regulation of cardiac remodeling and vascular smooth muscle cell differentiation, but little is known about its role in atherosclerosis.

**Methods:**

We tested the hypothesis that DKK3 is atheroprotective using both epidemiological and experimental approaches. Blood DKK3 levels were measured in the Bruneck Study in 2000 (n=684) and then in 2005 (n=574). *DKK3*-deficient mice were crossed with *apolipoprotein E*^-/-^ mice to evaluate atherosclerosis development and vessel injury-induced neointimal formation. Endothelial cell migration and the underlying mechanisms were studied using in vitro cell culture models.

**Results:**

In the prospective population-based Bruneck Study, the level of plasma DKK3 was inversely related to carotid artery intima-media thickness and 5-year progression of carotid atherosclerosis independently from standard risk factors for atherosclerosis. Experimentally, we analyzed the area of atherosclerotic lesions, femoral artery injury-induced reendothelialization, and neointima formation in both *DKK3*^*-/-*^*/apolipoprotein E*^*-/-*^ and *DKK3*^*+/+*^*/apolipoprotein E*^*-/-*^ mice. It was demonstrated that DKK3 deficiency accelerated atherosclerosis and delayed reendothelialization with consequently exacerbated neointima formation. To explore the underlying mechanisms, we performed transwell and scratch migration assays using cultured human endothelial cells, which exhibited a significant induction in cell migration in response to DKK3 stimulation. This DKK3-induced migration activated ROR2 and DVL1, activated Rac1 GTPases, and upregulated JNK and c-jun phosphorylation in endothelial cells. Knockdown of the ROR2 receptor using specific siRNA or transfection of a dominant-negative form of Rac1 in endothelial cells markedly inhibited cell migration and downstream JNK and c-jun phosphorylation.

**Conclusions:**

This study provides the evidence for a role of DKK3 in the protection against atherosclerosis involving endothelial migration and repair, with great therapeutic potential implications against atherosclerosis.

## Abstract 2

Supplemental Digital Content is available in the text.

**Clinical Perspective**

**What Is New?**

- We found that the plasma level of dickkopf-related protein 3 (DKK3), a member of the dickkopf family, is negatively correlated with atherosclerosis in human subjects.
- We demonstrated that DKK3 promotes reendothelialization in murine models of atherosclerosis and wire-induced femoral artery injury, thus revealing its atheroprotective role.
- We explored the mechanism of DKK3-induced endothelial cell migration (ie, by noncanonical Wnt signaling pathway).

**What Are the Clinical Implications?**

- The present finding of an inverse association between plasma DKK3 level and atherosclerosis may provide a novel biomarker for endothelial integrity and repair.
- DKK3 exhibits atheroprotective characteristics, which may bear clinical potential for the treatment of atherosclerosis.

The dickkopf-related protein (DKK) family, composed of DKK1, 2, 3, 4, and Soggy, is a group of secreted glycoproteins, of which DKK3 is highly expressed in endothelium and muscles.^1–4^ DKK3 appears to have a decisive function in myogenic cell fate because it is also highly expressed in different skeletal muscle subtypes.^5^ DKK3 has also been established as a potential tumor biomarker expressed in many cancer cell lines and an effective tumor suppressor in numerous human cancers.^6–8^ It has been reported that DKK3 plays a role in promoting angiogenesis in different types of tumors.^9,10^ Recently, several studies have discovered that DKK3 prevented the progression of cardiac hypertrophy^11,12^ and was also involved in vascular smooth muscle cell differentiation.^2,4^ However, the involvement of DKK3 in vascular diseases such as atherosclerosis remains unknown.

Atherosclerosis is characterized by endothelial dysfunction, inflammation, progressive lipid deposition, and vessel stiffness, with potential complications such as myocardial infarction or stroke.^13,14^ The endothelium, as a crude restrictive barrier of the vessel wall, can protect the vessel from inflammation. Once endothelial cells are impaired, they will become the initial sensors of a complex cascade of events.^15^ Many studies have demonstrated that the underlying pathophysiology of atherosclerosis is initiated by endothelial dysfunction,^16^ which is caused by physical or chemical offenses such as hypertension,^17^ shear stress of disturbed laminar flow,^18,19^ reactive oxygen species in the circulation,^20^ decreased nitric oxide bioactivity,^21^ hyperlipidemia, and hyperglycemia.^22^ These factors can directly or indirectly induce endothelial dysfunction/death in arteries,^23^ followed by cell regeneration in situ.^24^ In this process, neighboring endothelial cells have been proven to contribute to reendothelialization by migration and proliferation.^25^ In previous studies, vascular endothelial growth factor has been identified as a potent soluble factor for acceleration of reendothelialization and prevention of neointima formation.^26,27^ However, whether some novel soluble molecules are also playing a role in endothelial repair remains under investigation. In the present study, we take advantage of relevant human samples, transgenic animals, and in vitro cell biology models to elucidate the potential impact of DKK3 in atherosclerosis. In humans, we observed an inverse correlation between blood DKK3 level and development of atherosclerosis. In addition, we utilized genetic knockout mouse models combined with apolipoprotein E (ApoE)^*-/-*^ mouse to assess the effects of DKK3 on atherosclerosis, reendothelialization, and neointima formation after femoral artery injury. We found that DKK3 promoted reendothelialization and inhibited lesion formation in DKK3^+/+^ApoE^*-/-*^ mice. Our in vitro studies also revealed that DKK3 can induce endothelial cell migration by noncanonical Wnt signaling pathway.

## Methods

An expanded Methods is available in the online-only Data Supplement.

### Study Population

Population recruitment was performed as part of the prospective community-based Bruneck Study.^28,29^ The survey area was located in the north of Italy (Bolzano Province). Special features of the study design and protocol have been described previously in detail^28–30^ and are provided in the online-only Data Supplement. The current study focused on the evaluation in 2000 (n=684) and follow-up between 2000 and 2005. The appropriate ethics committees approved the study protocol, and all study subjects gave their written informed consent before entering the study.

### Enzyme-Linked Immunosorbent Assay (ELISA) for Plasma DKK3

The levels of DKK3 in human plasma were detected using an R&D DKK3 ELISA kit (R&D, DY1118). DKK1 levels were measured in serum with a commercial ELISA (Biomedica): Intra- and interassay coefficients of variation were low at 3% each, and the lower detection limit was 1.6 pmol/L.

### Animals

All animal experiments were performed according to the protocols approved by the Institutional Committee for the Use and Care of Laboratory Animals. ApoE^*-/-*^ mice were purchased from Jackson Laboratory. DKK3^*-/-*^ mice were generated as described previously.^31^ Three genotypes of DKK3^*-/-*^, DKK3^-/+^, and DKK3^+/+^ mice were identified using PCR (primers: 5-GATAGCTTTCCGGGACACAC-3, 5-TCCATCAGCTCCTCCA CCTCT-3, 5-TAAGTTGGGTAACGCCAGGGT-3). ApoE^*-/-*^ mice were crossed with DKK3^*-/-*^ mice in our laboratory, and heterozygous offsprings were mated to produce ApoE^*-/-*^ mice lacking DKK3 (DKK3^*-/-*^ ApoE^*-/-*^). The genetic background of all mice used in the present study was C57BL/6.

### Creation of Chimeric Mice

The procedure used for creating chimeric mice was similar to previously described.^32^ In brief, bone marrow transplantation was carried out on the DKK3^+/+^ and DKK3^*-/-*^ mice separately. Bone marrow cells were obtained from the femurs and tibias of either DKK3^+/+^ or DKK3^*-/-*^ mice (donors) and injected (1x10^7^ cells in 0.2 mL) into the tail veins of the 6- to 8-week-old DKK3^*-/-*^ or DKK3^+/+^ mice (recipients), which received lethal irradiation (950 Rads) before. The measurement of DKK3 level in peripheral blood was performed 3 weeks after bone marrow transplantation.

### Tissue Harvesting and Lesion Analysis

Mice were anesthetized by intraperitoneal injection of pentobarbital atrium (50 mg/kg b.w.). Blood was obtained from inferior vena cava for lipid analysis. The heart was harvested intact and stored immediately in liquid nitrogen, and the whole length of the aorta was stored in formalin at 4°C. Then 8-μm-thick frozen sections were obtained from the heart and stained with oil Red O as described elsewhere.^33^ Aortas were opened longitudinally and fixed on a silicon bed with stainless steel pins (Fine Science Tool) with the intima exposed. Oil Red O staining was performed. Lesion areas were measured and quantified using a computer software AxioVision as described previously.^33a^

### Transwell Chemotaxis Assay

Migration chemotaxis assay was performed by applying 24-well Boyden chambers with 8-µm pore size polycarbonate membranes (Corning) as described previously.^34^ Human umbilical vein endothelial cells (HUVECs) were seeded onto the upper chamber at 1x10^5^ cells in 0.1% FBS EBM-2 basal medium, while the bottom chamber contained either 0.1% FBS EBM-2 basal medium with indicated concentrations of recombinant human DKK3 or Adeno-DKK3-HA/Adeno-CMV null overexpressed CHO cells supernatant. 0.1% FBS EBM-2 basal medium served as negative control for the comparison with recombinant human DKK3. After incubation for 6 hours at 37°C, the cells remaining on the upper side of the filters were removed by a cotton swab. The migrated cells on the underside of the membrane were fixed with 4% paraformaldehyde before staining with 0.1% crystal violet solution for 15 minutes. Data were expressed as the fold of migrated HUVECs compared to their corresponding controls.

### Statistical Analysis

#### Population Study

The data were analyzed using the SPSS 24 software package. Levels of variables according to DKK3 tertile groups were presented as mean values ± SD or as medians with corresponding 25th and 75th percentiles (continuous variables) and percentages (dichotomous variables). Associations between DKK3 level (predictor variable) and vascular risk factors, lifestyle and demographic variables, intima-media thickness (IMT), and atherosclerosis progression were assessed using linear and logistic regression analysis. Levels of C-reactive protein and triglycerides were log_e_-transformed to satisfy the assumption of normality and constant variance of the residuals. The multivariate models focusing on IMT or atherosclerosis progression included the following covariates: age (years), sex (female, male), smoking (cigarettes per day), hypertension, high- and low-density lipoprotein cholesterol, triglycerides, high-sensitivity C-reactive protein, creatinine, body mass index, waist-to-hip ratio, chronic infections, fasting glucose, and physical activity (sports score). A 2-sided *P* value <0.05 was considered significant.

#### In Vivo and in Vitro Studies

Data for in vivo and in vitro studies are presented as the mean±standard error of the mean of ≥3 separate experiments. The analysis was performed using Graphpad Prism V.6 (GraphPad Software) using Student’s *t* test between 2 groups and 1-way analysis of variance followed by Dunnett’s multiple comparison test for more than 2 groups. A *P* value <0.05 was considered significant.

## Results

### Plasma DKK3 Level Is Negatively Correlated With Atherosclerosis in the Population-Based Bruneck Study

**Figure 1.**
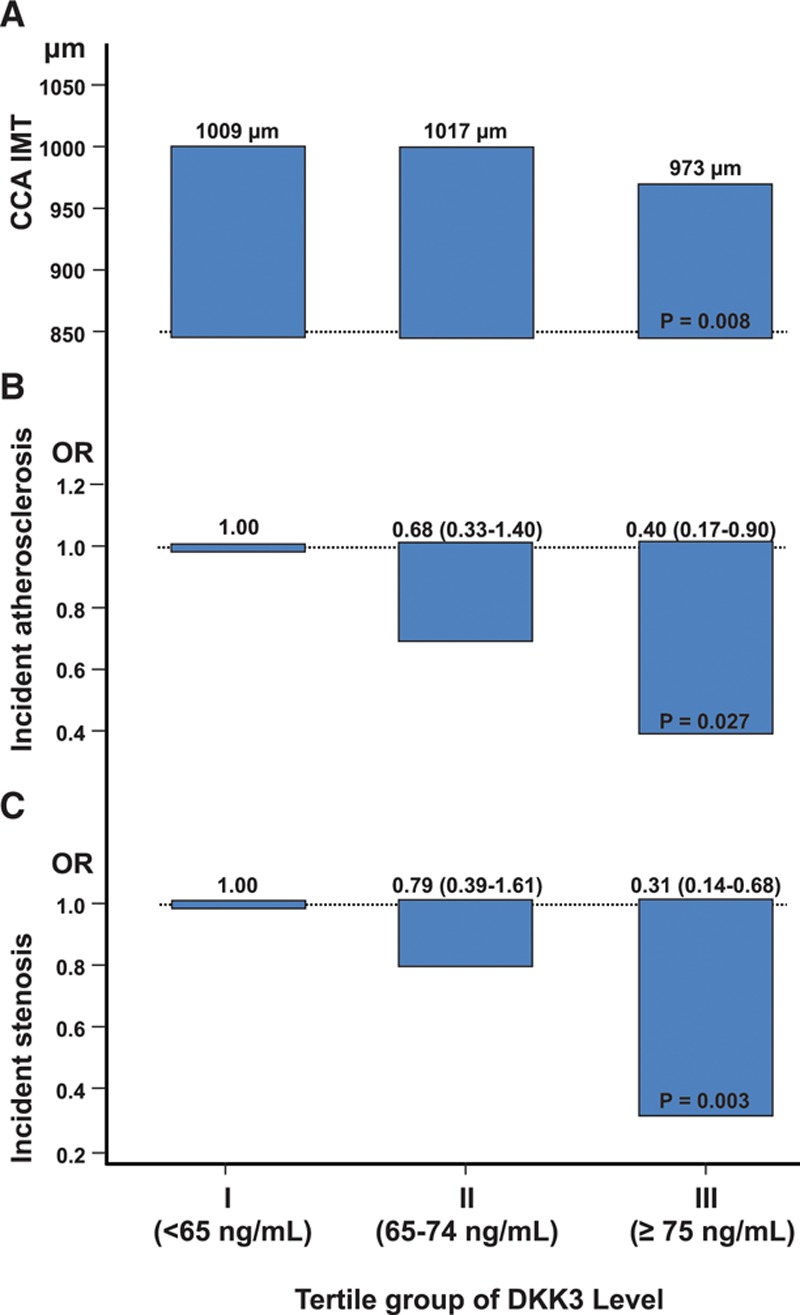
**Plasma DKK3 level is inversely correlated with atherosclerosis in the prospective population-based Bruneck Study. A**, Age- and sex-adjusted mean common carotid artery intima-media thickness (CCA-IMT) was grouped according to DKK3 tertile groups. Odds ratios of incident carotid atherosclerosis (early atherogenesis) (**B**) and incident carotid stenosis manifesting between 2000 and 2005 (advanced atherogenesis) (**C**) according to baseline (2000) DKK3 tertile groups. The bottom tertile group served as the reference category. Analyses were adjusted for age and sex. DKK3 indicates dickkopf-related protein 3.

The Bruneck study is a prospective survey on atherosclerosis and its risk factors, as well as protective mechanisms against it.^28,29^ Plasma was collected from a random sample of the general community. A total of 611 samples from the year 2000 and 554 samples from the year 2005 were analyzed for DKK3 concentrations using ELISA. DKK3 levels in both assessments were highly correlated, indicating a low intraindividual variability of a 5-year interval (Spearman’s rank correlation coefficient *r*=0.500) (online-only Data Supplement Figure I). Population characteristics according to DKK3 tertile groups are depicted in the Table. Age increased across DKK3 tertile groups (*P*<0.001). After adjustment for age and sex, several standard and emerging cardiovascular risk factors showed association with DKK3 level (creatinine, body mass index, waist-to-hip ratio, and fasting glucose), and all these associations were inverse (Table). It is important to note that the common carotid artery (CCA) IMT, a surrogate marker of early vessel pathology, was reduced in the top DKK3 tertile group (*P*=0.008) (Figure 1A). A gradual decrease was also seen in the risk of incident atherosclerosis (incident plaques, n=59 out of 259 subjects free of carotid atherosclerosis at baseline; Figure 1B) and carotid artery stenosis (advanced plaques, n=63 out of 332 subjects with preexisting carotid atherosclerosis; Figure 1C) across DKK3 tertile groups (*P*<0.05 each).

**Table.**
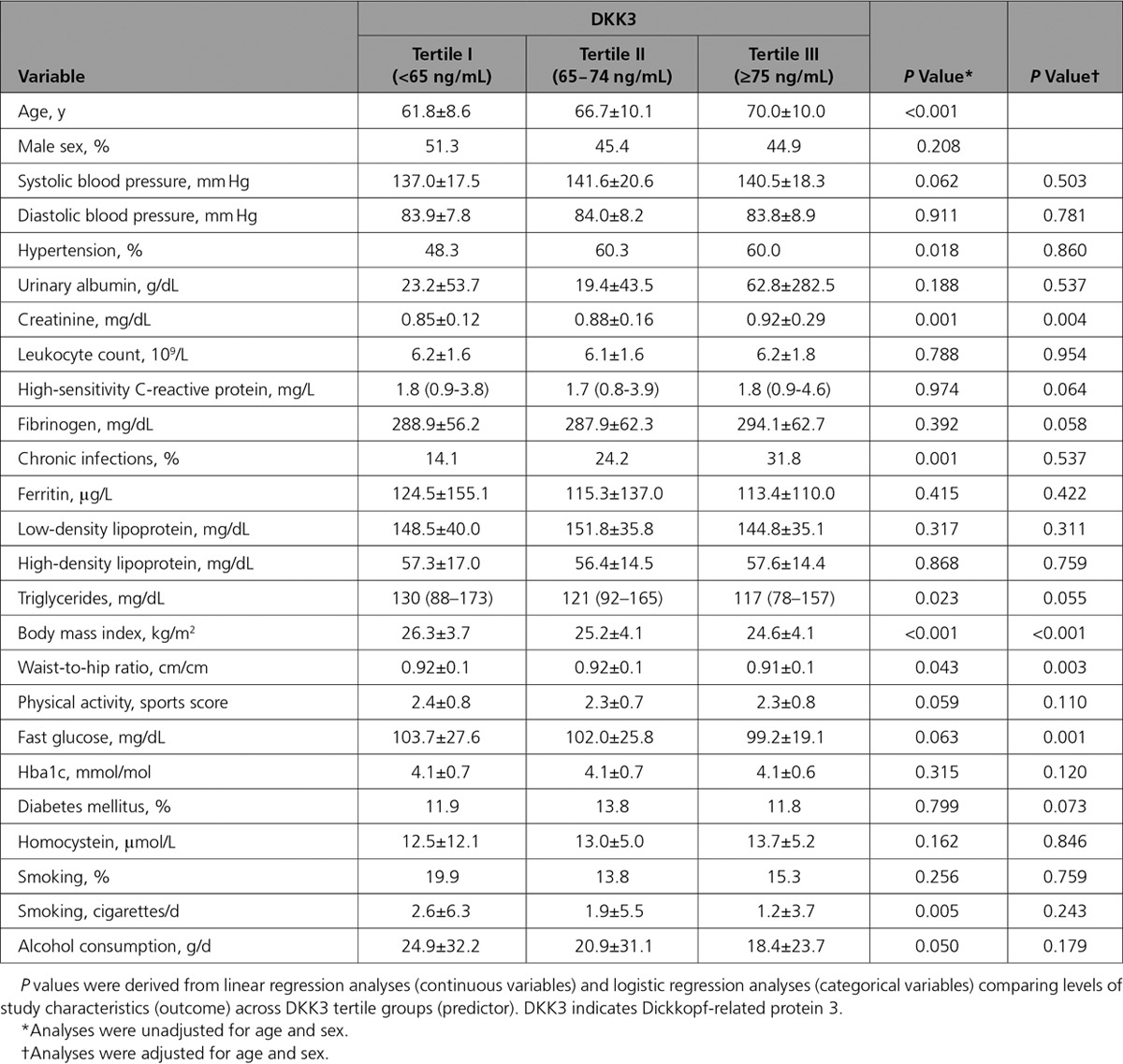
Levels of Study Characteristics According to DKK3 Tertile Groups in the Bruneck Study

We next calculated the odds ratios of incident atherosclerosis and stenosis for a 1-SD unit higher DKK3 level (logistic regression analysis, *P*=0.035 and 0.004, model 1, online-only Data Supplement Table I) and confirmed the associations to be independent of a broad panel of established and putative vascular risk factors, including those significantly related to DKK3 level in Table (model 2, online-only Data Supplement Table I). Similar findings were obtained with CCA-IMT (online-only Data Supplement Table I). Overall, strong evidence exists of an inverse association between DKK3 level and both early and advanced stages of atherosclerosis. To scrutinize the correlation between DKK3 and other circulating factors in the blood, granulocyte-colony stimulating factor, matrix metallopeptidase 9, vascular endothelial growth factor, stromal cell-derived factor 1, soluble receptor activator of nuclear factor kappa β ligand, osteoprotegerin, and angiogenic cells levels were measured. Stromal cell-derived factor 1 was significantly related to DKK3 (*P*=0.021, adjusted for age and sex) (online-only Data Supplement Table II).

It is interesting to note that the level of DKK1, another member of the Dickkopf family, showed a significant positive association with CCA-IMT (age- and sex-adjusted regression coefficient [95% confidence interval] for a 1-SD unit higher DKK1 level, 0.031 [0.008–0.054]). The inverse association between DKK3 and CCA-IMT was more pronounced in subjects with high (≥44.1 pmol/L; ie, ≥median) DKK1 level (age- and sex-adjusted regression coefficient [95% confidence interval] for a 1-SD unit higher DKK3 level, -0.018 [-0.037 to 0.001]) than in those with low (<44.1 pmol/L) DKK1 level (age- and sex-adjusted regression coefficient [95% confidence interval] for a 1-SD unit higher DKK3 level, -0.011 [-0.027 to 0.004]), but this interactive effect between DKK1 and DKK3 did not reach statistical significance (*P*_interaction_=0.17).

#### Deficiency of DKK3 Promotes Atherosclerosis in Mice

**Figure 2.**
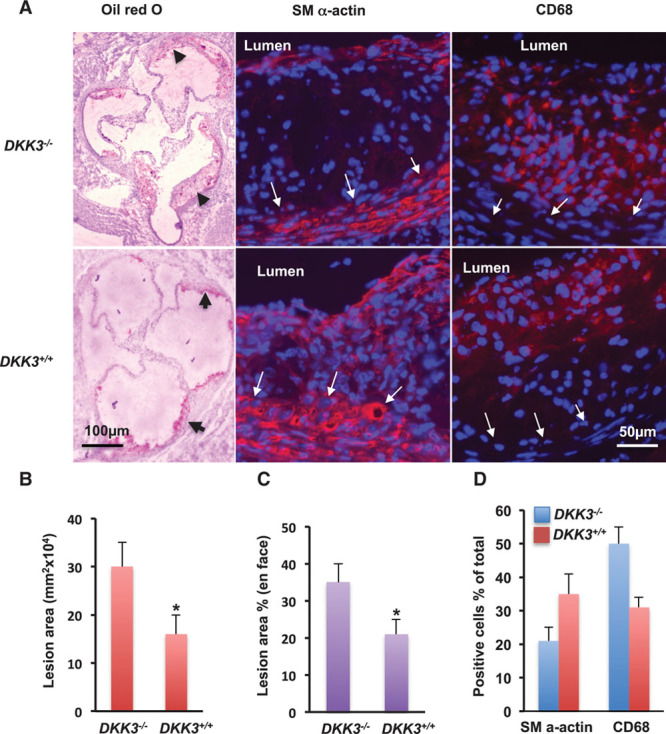
**Atherosclerotic lesions in *DKK3***^*-/-*^*/ApoE*^*-/-*^ **mice.** Mice receiving normal chow diet were euthanized at 20 weeks old, and the heart and aorta were harvested. The aortic root was sectioned, and aortas were mounted and stained with oil Red O. Lesional areas in the aortic sinus and surface were measured and quantified as described in the Methods. The sections were immunostained with anti-α-smooth actin and CD68 antibodies, respectively. Positive cells were quantified under the microscope. **A**, Representative pictures of aortic sinus sections stained for oil Red O, α-smooth actin to identify smooth muscle cells, and CD68 to identify macrophages; measurement of lesions size on aortic sinus sections (**B**), on en face stained-lesion areas (**C**; % of total surface areas), and quantification of αSMA and CD68 positive cells (**D**; % of total cells). *Significant difference between *DKK3*^*-/-*^*/ApoE*^*-/-*^ and *DKK3*^*+/+*^*/ApoE*^*-/-*^ groups, *P<0.05 (n=11). ApoE indicates apolipoprotein E; and DKK3, dickkopf-related protein 3.

To investigate the role of DKK3 in the development of atherosclerosis, we crossed *DKK3*-deficient mice^35^ with *ApoE*^*-/-*^ animals to generate *DKK3*^*-/-*^ *ApoE*^*-/-*^ mice (online-only Data Supplement Figure II). As observed with the measurement of atherosclerotic lesions by en face staining (oil red O) of aortas (Figure 2C and online-only Data Supplement Figure III), cross-sectional analysis of aortic root samples in male mice fed a normal chow diet for 16 weeks revealed smaller lesion in *DKK3*^*+/+*^*/ApoE*^*-/-*^ than in *DKK3*^*-/-*^*/ApoE*^*-/-*^ mice (Figure 2A and 2B). Analysis by immunofluorescence showed a significant increase of αSMA staining in lesions from *DKK3*^*+/+*^*/ApoE*^*-/-*^ mice (Figure 2A and 2D) compared with *DKK3*^*-/-*^ *ApoE*^*-/-*^, suggesting that these smaller lesions were more stable and less advanced. Furthermore, a staining against CD68 marker revealed a reduction in the number of lesional macrophages in *DKK3*^*+/+*^*/ApoE*^*-/-*^ mice (Figure 2A and 2D). These data together suggest that DKK3 could have a protective role against atherosclerosis in *ApoE*^*-/-*^ mice.

### DKK3 Deficiency Leads to Endothelial Dysfunction in *ApoE*^*-/-*^ Mice

**Figure 3.**
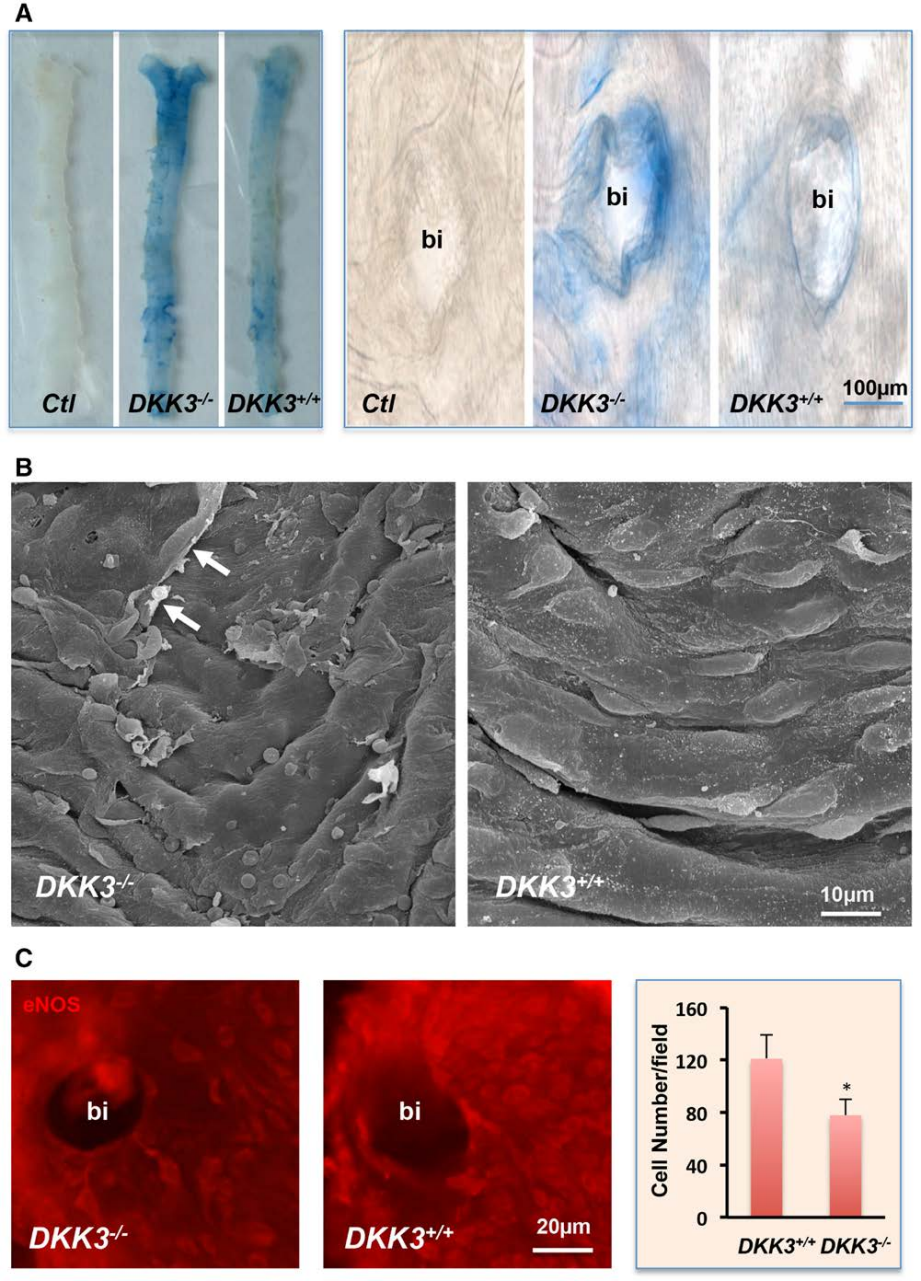
**Increased endothelial damage in *DKK3***^***-/-***^***/ApoE***^***-/-***^ **mice. A**, Evans blue dye leaking study. Ten-week-old *DKK3*^*-/-*^*/ApoE*^*-/-*^ and *DKK3*^*+/+*^*/ApoE*^*-/-*^ mice received an injection of 1% Evans blue dye by the tail vein. Mice were euthanized, and aortas were harvested and washed. Blue areas, representing the damaged area on the surface of aortas were observed. **B**, Scanning electron microscopy analysis of aortic samples from *DKK3*^*-/-*^*/ApoE*^*-/-*^ and *DKK3*^*+/+*^*/ApoE*^*-/-*^ mice. Arrows indicate the damaged cell. **C**, En face preparation of the vessel was stained for endothelial marker eNOS and visualized after incubation with secondary Cy3 conjugated antibody. Quantitative data analysis of the number of endothelial eNOS-positive cells around the bifurcation (bi) areas are presented in the graph (n=6). *P<0.05, indicating significant difference between the 2 groups. ApoE indicates apolipoprotein E; DKK3, dickkopf-related protein 3; and eNOS, endothelial nitric oxide synthase.

It is well established that endothelial cells play an essential role in homeostasis of the vascular wall.^36^ To investigate the possible mechanism associated with the accelerated atherosclerosis in *DKK3*^*-/-*^*/ApoE*^*-/-*^ mice, we first analyzed the integrity of the endothelium by injecting Evans blue in *DKK3*^*-/-*^*/ApoE*^*-/-*^ and *DKK3*^*+/+*^*/ApoE*^*-/-*^ mice. The aortas harvested from *DKK3*^*-/-*^*/ApoE*^*-/-*^ mice exhibited a larger blue area, indicating more endothelial damage (Figure 3A). Scanning electron microscopy analysis demonstrated apparent endothelium loss in the aortas from *DKK3*^*-/-*^*/ApoE*^*-/-*^ mice (Figure 3B). To further confirm the impaired endothelial integrity in *DKK3*^*-/-*^*/ApoE*^*-/-*^ mice, immunofluorescence staining of endothelial nitric oxide synthase was performed. A significant decrease in the number of endothelial nitric oxide synthase-positive cells was observed in *DKK3*^*-/-*^*/ApoE*^*-/-*^ mice (Figure 3C). These data indicate that DKK3-deficient mice display increased endothelium damage, suggesting a protective role of DKK3 on the endothelium in the context of atherosclerosis.

#### DKK3 Deficiency Delays Reendothelialization and Aggravates Neointima Formation in Wire-Injured Murine Femoral Arteries

**Figure 4.**
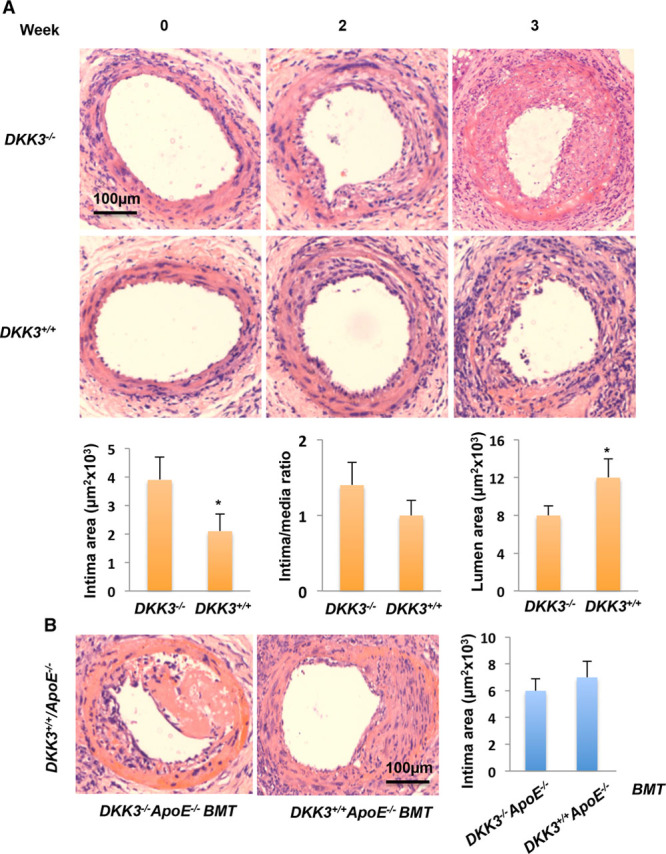
**Increased neointimal lesions in *DKK3***^***-/-***^***/ApoE***^***-/-***^ **mice after vessel injury**. Femoral arteries were wire injured, harvested at different times after surgery, and cross-sectioned for morphological analysis. **A**, Representative hematoxylin and eosin-stained sections of femoral arteries from *DKK3*^***-/-***^*/ApoE*^***-/-***^ and *DKK3*^*+/+*^*/ApoE*^***-/-***^ mice (**top**). Means±SEM from 6 mice for each group of the neointimal area, neontima/media ratio, and luminal area are presented (**bottom**). *P<0.01, indicating a significant difference between the two groups. **B**, Representative hematoxylin and eosin-stained sections of femoral arteries from a chimeric mouse, which is *DKK3*^*+/+*^*/ApoE*^*-/-*^ mouse receiving *DKK3*^*-/-*^*/ApoE*^*-/-*^ or *DKK3*^*+/+*^*/ApoE*^*-/-*^ bone marrow after irradiation 3 weeks after vessel injury. The graph shows means±SEM of lesion areas (n=6). No significant difference was noted between the 2 groups. ApoE indicates apolipoprotein E; BMT, bone marrow transplantation; and DKK3, dickkopf-related protein 3.

To test the hypothesis that DKK3 is involved in endothelium recovery after injury, we performed wire injury in femoral arteries of *DKK3*^*-/-*^*/ApoE*^*-/-*^ and littermate *DKK3*^*+/+*^*/ApoE*^*-/-*^ mice. Reendothelialization of the arteries was quantified 1 week after injury by en face staining of endothelial nitric oxide synthase. Endothelial cells were recovered by 80% after 1 week in *DKK3*^*+/+*^mice. In contrast, reendothelialization in *DKK3*^*-/-*^ mice was only ≈50% recovery 1 week after injury (online-only Data Supplement Figure IV). Subsequent neointimal hyperplasia was dramatically aggravated in *DKK3*^*-/-*^ mice 2 and 3 weeks after injury, as demonstrated by increased intima area and reduced lumen area (Figure 4A). To test whether the expression of DKK3 in hematopoietic cells contributed to this process, we performed bone marrow transplantation experiments. In the wild-type chimaera, DKK3 level in mice serum was significantly increased after 2 weeks despite transplantation with *DKK3*^*-/-*^ bone marrow when compared with a nontransplanted wild-type mouse control, indicating that DKK3 is mostly released from nonbone marrow tissues (online-only Data Supplement Figure V). *ApoE*^*-/-*^*/DKK3*^*+/+*^ chimeric mice transplanted with *DKK3*^*-/-*^ bone marrow showed comparable neointimal hyperplasia to *ApoE*^*-/-*^*/DKK3*^*+/+*^ mice with wild-type bone marrow 3 weeks after femoral artery wire injury (Figure 4B), indicating that DKK3 expression in hematopoietic-derived cells does not contribute to protection from atherosclerosis and neointima formation postinjury.

#### Exogenous DKK3 Induces Endothelial Cell Migration

**Figure 5.**
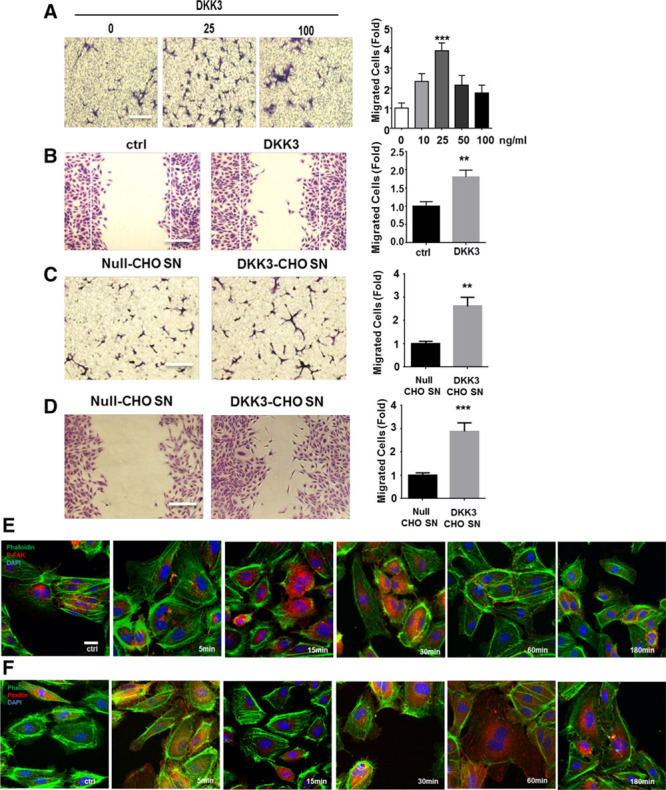
**DKK3 can induce endothelial cell migration.** Chemotaxis of human umbilical vein endothelial cells (HUVECs; 1×10^5^ cells/well) across 8.0-μm transwells toward either human recombinant DKK3 (**A**) or adeno-DKK3-overexpressed CHO supernatant (**C**) was counted 6 hours after crystal violet staining; 0.1% FBS EBM-2 medium or adeno-CMV null-overexpressed CHO supernatant was used as controls, respectively (n=5, Bars, 100 µm). **B**and **D**, Scratch-wound assay was performed on HUVECs. The chemotaxis index of each quantification was defined by the average of 9 fields of view from each well and was presented as fold increase compared with the corresponding controls (n=5). All graphs are shown as mean±SEM. **P<0.01; ***P<0.001. Null-CHO SN indicates adeno-null-overexpressed CHO supernatant; and DKK3-CHO SN, adeno-DKK3-overexpressed CHO supernatant (Bars, 100 µm). **E**and **F**, HUVECs were treated with human recombinant DKK3 for indicated time points before immunofluorescence staining of phosphorylated FAK and paxillin. Ctrl indicates control; and p-FAK, phosphorylated FAK (Bars, 50 µm). DKK3 indicates dickkopf-related protein 3.

To study whether DKK3 can directly stimulate endothelial cell migration in vitro, both transwell and scratch-wound healing migration assays were performed. Data showed that endothelial cell (HUVEC) migration was significantly induced by human recombinant DKK3 and peaked at 25 ng/mL (Figure 5A and 5B). Similarly, mouse DKK3 induced mouse lung endothelial cell migration, indicating a conserved mechanism (online-only Data Supplement Figure VIA and VIB). To further investigate whether the glycosylated form of DKK3 contributes to endothelial cell migration, CHO cell lines, which do not express native DKK3, were transduced with human HA-tagged DKK3 adenovirus to produce the protein. Initially, quantitative polymerase chain reaction and ELISA analysis confirmed DKK3 expression in both CHO cell lysates and their supernatant in a dose-dependent manner (online-only Data Supplement Figure VIC and VID). In addition, Western blot analysis was performed to detect glycosylated DKK3 (65KD) in CHO cell supernatant and deglycosylated DKK3 (50KD) in cell lysate (online-only Data Supplement Figure VIE and VIF). Furthermore, the supernatant of DKK3 adenovirus-induced CHO cells (ADV-DKK3-SN) was utilized in migration assays to confirm endothelial cell movement (Figure 5C and 5D). Together these results suggested that glycosylated DKK3 present in ADV-DKK3-SN was responsible for the effects observed in endothelial cell migration. To rule out the possibility that residual adenoviral particles present in ADV-DKK3-SN could lead to overexpression of DKK3 in endothelial cells and affect cells’ migration, a quantitative polymerase chain reaction analysis was performed and showed that the increase of DKK3 expression in ADV-DKK3-SN-treated endothelial cells was negligible in comparison with the induction of DKK3 expression in endothelial cells directly transduced with DKK3 adenovirus (online-only Data Supplement Figure VIG through VII). Moreover, endothelial migration on DKK3 protein stimulation was also confirmed by phospho-FAK and paxillin staining (Figure 5E and 5F). A murine subcutaneous Matrigel plug assay showed that DKK3 also induced endothelial cell migration in vivo (online-only Data Supplement Figure VII). In contrast, BrdU cell proliferation and Annexin V apoptosis assays on HUVECs incubated with the recombinant DKK3 protein or ADV-DKK3-SN revealed that DKK3 had no effect on cell proliferation or apoptosis (online-only Data Supplement Figure VIIIA and VIIIB). Taken together, these results support the notion that exogenous DKK3 (especially glycosylated DKK3) significantly induces endothelial cell migration.

#### DKK3 Induces Endothelial Cell Migration Through the Wnt Pathways

**Figure 6.**
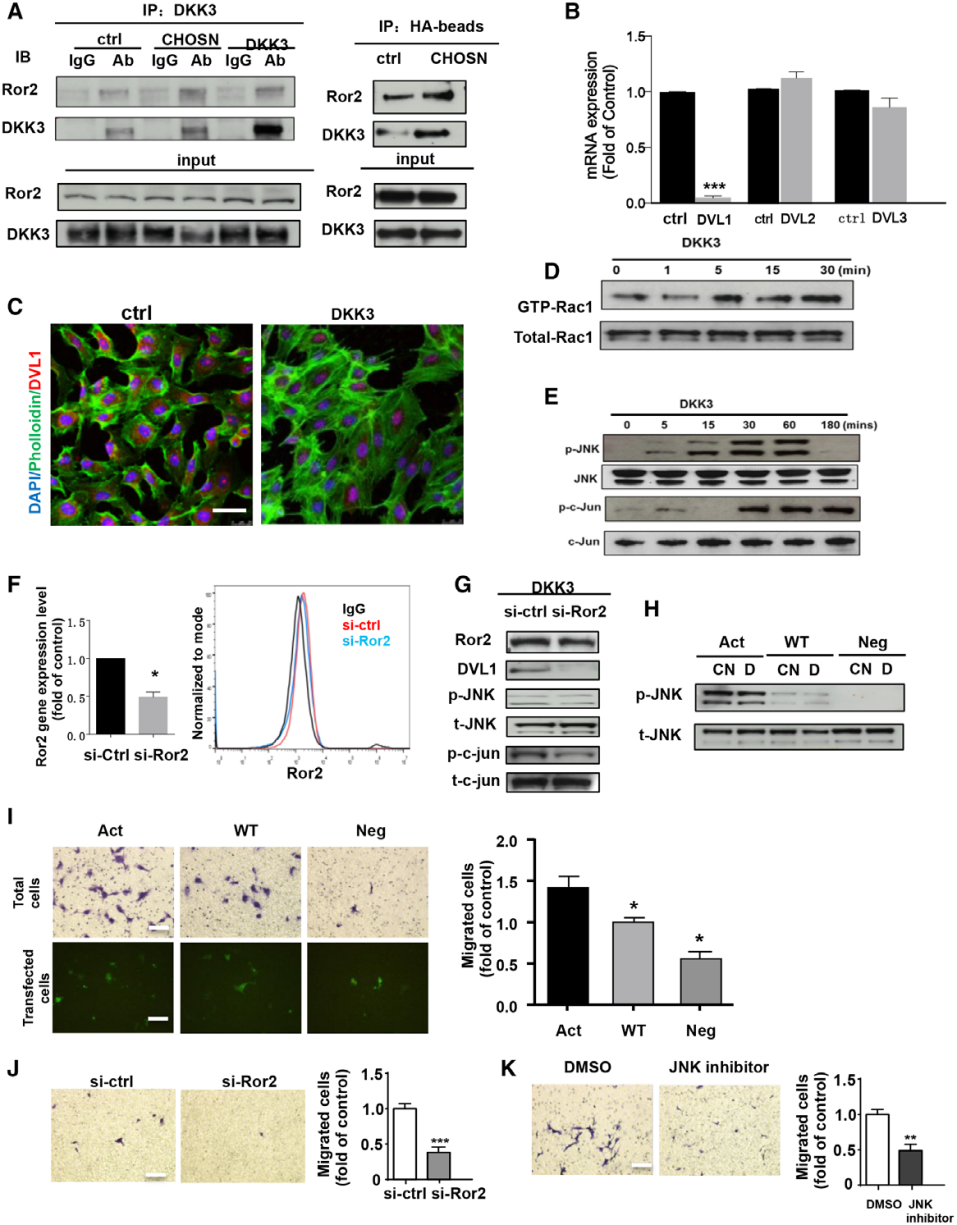
**DKK3 induces endothelial cell migration by ROR2-Dvl1-Rac1-JNK signaling pathway. A**, Western blot analysis showed the binding of ROR2 and DKK3 after immunoprecipitation of either DKK3 or HA-binding protein in HUVECs stimulated with DKK3 or DKK3-CHO SN. **B**, The real-time quantitative polymerase chain reaction showed the fold changes in mRNA levels of DVL1, DVL12, and DVL13 after DKK3 treatment. **C**, Immunofluorescence staining showed the translocation of ROR2 and DVL1 in the nucleus after DKK3 treatments. **D**, Pull-down assays were performed on HUVECs treated with DKK3 from 1 to 30 minutes to analyze GTP-Rac1 activation. **E**, Western blot analysis was performed on DKK3-treated HUVECs for the detection of JNK and c-jun phosphorylation and their total expression. **F**, The gene and protein levels of ROR2 were analyzed by quantitative polymerase chain reaction and fluorescence-activated cell sorting after knockdown by ROR2 siRNA. Western blotting showed the levels of ROR2, DVL1 proteins, and JNK and c-jun phosphorylation in HUVECs after either control siRNA or ROR2 siRNA transfection (**G**) or phosphorylated JNK and c-jun levels in HUVECs after (Continued ) **Figure 6 Continued.** transfection of constitutive mutants of Rac1 (**H**). Transwell assay was performed on HUVECs that were either transfected with constitutive mutants of Rac1 (**I**) or ROR2 siRNA (control) (**J**) before migration toward DKK3 for 6 hours with either crystal violet staining or direct fluorescence observation of plasmid-transfected cells. **K**, Transwell assay was performed on HUVECs that migrated toward DKK3 in the presence of JNK inhibitor (SP600125) for 6 hours. All the blots shown are representative of 3 separate experiments. All graphs are shown as mean±SEM (n=3). *P<0.05; **P<0.01; ***P<0.001 (Bars, 100 µm). Ab indicates antibody; act, constitutively active mutant; CHOSN, CHO cells derived-supernatant; CN, supernatant; ctrl, control; D, DKK3; DKK3, dickkopf-related protein 3; DMSO, dimethyl sulfoxide; IgG, immunoglobulin G; neg, constitutively negative mutant; p-c-jun, phosphorylated c-jun; p-JNK, phosphorylated JNK; si-ctrl, control siRNA; si-Ror2, Ror2 siRNA; t-c-jun, total c-jun; t-JNK, total JNK; and wt, constitutively wt mutant.

β-catenin has been implicated in the regulation of several DKK3 functions through the Wnt/β-catenin signaling pathway.^3^ However, in DKK3-treated endothelial cells, β-catenin was not activated as indicated by Western blot analysis or immunofluorescence staining (online-only Data Supplement Figure IXA through IXC). Distinct from a role of the canonical Wnt/β-catenin pathway in the regulation of cell proliferation and development,^37^ the noncanonical Wnt pathway is involved in cell polarity^38^ and convergent extension movements.^39^ Recent studies have indicated that the tyrosine kinase receptor ROR2 plays an important role in the noncanonical Wnt pathway to mediate cell migration.^40–42^ To investigate whether DKK3 induces cell migration through the β-catenin-independent noncanonical Wnt-PCP pathway, we first performed a coimmunoprecipitation analysis, which revealed the binding of DKK3 to ROR2 in endothelial cells after stimulation with either recombinant DKK3 or DKK3-CHO-SN (Figure 6A). This result indicates that transmembrane receptor ROR2 could be a potential binding site for DKK3. As previous studies have demonstrated, members of the Dishevelled family can mediate the Wnt-PCP signaling pathway after activation of ROR2. Hence, we further investigated Dvl1, 2, 3 gene expression levels and found that only Dvl1 displayed a 5-fold reduction on stimulation with DKK3 after 6 hours (Figure 6B). Further immunofluorescence staining of DVL1 revealed its translocation from cytoplasm to the nucleus after DKK3 treatments for 6 hours (Figure 6C).

To study the downstream signaling pathways activated after the binding of DKK3 to ROR2, we measured the level of GTP-Rac1 and GTP-RhoA using pull-down assays. The level of GTP-Rac1 (Figure 6D) but not GTP-RhoA (online-only Data Supplement Figure X) was significantly upregulated by both recombinant human DKK3 and CHO supernatant, which indicated that Rac1 but not RhoA can be activated by DKK3. A time course Western blotting analysis showed phosphorylation of JNK, and c-jun occurred as early as 5 minutes after treatment with DKK3 in endothelial cells (Figure 6E). siRNA-mediated knockdown of ROR2 in DKK3-treated HUVECs (Figure 6F) significantly reduced downstream DVL1 protein level, JNK, and c-jun phosphorylation (Figure 6G) and inhibited cell migration, therefore placing ROR2 at the top of the signaling cascade mediating DKK3-induced migration (Figure 6J). Successful transfection of plasmids coding for enhanced green fluorescent protein-labeled constitutive Rac1 mutants were shown by quantitative fluorescence-activated cell sorting analysis (online-only Data Supplement Figure X). Moreover, phosphorylation of JNK was markedly upregulated in response to DKK3 treatment in cells transfected with constitutively active Rac1 but not with constitutively negative mutant (Figure 6H). It is important to note that the migratory abilities of endothelial cells were enhanced in Rac1 constitutively activated cells in response to DKK3 treatment (Figure 6I). Last, migration of endothelial cells toward DKK3 stimulation was markedly reduced in the presence of SP 600125 (an inhibitor of the JNK) (Figure 6K). Therefore, these data indicate that DKK3 induces endothelial cell migration by activating the Wnt-PCP signaling pathway by Rac1-JNK but not RhoA.

#### DKK3 Has No Effect on Leukocyte Migration

Leukocyte migration is critical for atherosclerosis development.^43,44^ Because DKK3 is a potent attractant for endothelial cell migration, it would be crucial to know whether it can also induce leukocyte migration toward the lesion area during atherogenesis. To investigate this issue, the different cell populations contained in peritoneal cells from mice after DKK3 injection into the abdominal cavity were analyzed by fluorescence-activated cell sorting and compared with the saline group (control). Unlike thioglycollate stimulation (positive control), in which cell number was markedly augmented, DKK3 treatment did not significantly increase the number of macrophages, T cells, or B cells in the total peritoneal cell population (online-only Data Supplement Figure XIA and XIB). Furthermore, in vitro migration assay revealed no significant differences in macrophage subpopulation migration toward various concentrations of mouse recombinant DKK3 (online-only Data Supplement Figure XIC). Thus, we conclude that DKK3 does not induce leukocyte recruitment and migration in vitro and in vivo.

## Discussion

In previous reports, the role of DKK3 as a potential tumor suppressor has been well studied in several human cancers.^45,46^ In recent years, DKK3 was found to act as a potent protector of cardiac hypertrophy by the Wnt signaling pathway,^11,12,47^ and it also has been associated with stem cell differentiation into vascular smooth muscle cells.^2,4^ In the present study, we found that plasma DKK3 level was inversely and independently associated with CCA-IMT and incident carotid atherosclerosis and stenosis over a 5-year follow-up, suggesting that DKK3 confers protection against both early and advanced stages of atherogenesis.

DKK3, as a secretory glycoprotein, can be released from a variety of tissues in mouse under physiological conditions, which explains the ubiquitous expression of DKK3 in vivo.^48^ In our study, we created chimeric mice models to further investigate which source of circulating DKK3 takes part in the protection from atherosclerosis and neointima formation. In the *DKK3*^-/-^ chimeric mouse with wild-type bone marrow model, the DKK3 level in serum was barely increased despite transplantation with wild-type bone marrow, indicating that DKK3 is mostly released from nonhematopoietic cells. It is interesting to note that the wild-type chimeric mice with *DKK3*^-/-^ bone marrow exhibited an even higher level of DKK3, implying that the bone marrow transplantation procedure may induce even more nonhematopoietic cell release of DKK3 into circulation. Given the evidence that endothelial cells express DKK3, the endothelium could be a source of circulating DKK3. When endothelial cells are damaged or dysfunctional, their ability to release DKK3 also might be decreased. Our findings support the notion that the lower levels of DKK3, which were found in the blood of patients with atherosclerosis, could be explained by lower DKK3 release because of endothelial dysfunction. Although the specific cellular and molecular mechanisms of DKK3 release remain unknown, further studies would be needed to clarify how DKK3 is released into the blood.

After being released into the blood, DKK3 may exert its effect on endothelial cells and subsequently the development of atherosclerosis. The endothelium is an indispensable barrier inside the vessel wall, and its integrity has been viewed as a determinant in atherogenesis, especially in the early stages.^49,50^ As a progressive chronic disease, once initiated by risk factors, atherosclerosis provokes a cascade of pathophysiological responses,^51^ including postangioplasty neointima formation and restenosis.^52^ In our study, different experimental models have confirmed that the endothelium was apparently dysfunctional or damaged in aortas of mice with DKK3 deficiency, suggesting that DKK3 plays a protective role in endothelial integrity. In *DKK3*^*+/+*^*/ApoE*^-/-^ mice, the atherosclerotic lesion area was smaller compared with *DKK3*^*-/-*^*/ApoE*^*-/-*^ mice. In the femoral artery wire injury model, DKK3 displayed properties of protection of endothelium integrity by accelerating the reendothelialization at the early stage as well as reduction of neointima formation at the late phase. The data derived from experimental models provide direct evidence that DKK3 could act as a chemokine-like protein in endothelial migration and thus be protective of atherosclerosis.

In the early stage of atherogenesis, endothelial regeneration is a critical protective mechanism to repair damaged cells and prevent the progression of atherosclerosis.^53,54^ As mentioned previously, an inverse correlation between blood DKK3 level and atherosclerosis in humans and a reduction of arterial reendothelialization after injury in the *DKK3*^*-/-*-^ mouse model have been observed. It is rational to investigate whether the effect of DKK3 on endothelial cell migration could contribute to endothelial repair. In vitro migration assays showed that exogenous DKK3 significantly induced endothelial cell migration. It was reported that DKK3 is a secreted glycoprotein with 4 potential *N*-glycosylation sites, and endogenous DKK3 will be glycosylated before its release into the supernatant.^3,48,55^ In our study, enhanced endothelial migration was induced by secreted and released DKK3 produced by Adeno-DKK3-CHO cells. The glycosylated form of DKK3 protein could be detected in the supernatant, suggesting that glycosylated DKK3 is the main actor on endothelial cell migration. DKK3-induced endothelial migration could play a part in atherogenesis, but other potential effects of DKK3 on the development of the disease are still unknown. Further studies would be needed to understand its roles in the pathogenesis of atherosclerosis.

Previous studies described that DKK3 was expressed in various tumor endothelial cells^9,10^ and that overexpression of DKK3 did not affect proliferation and migration of endothelial colony-forming cell.^10^ Similarly, our data also showed that DKK3 within the cell after Adeno-DKK3 transduction did not induce cell migration, indicating that DKK3 may need to interact with its receptor(s) on the cell surface to exert its effects on cell movements. As Wnt signaling pathway inhibitors, DKK family members DKK1, DKK2, and DKK4 were proved to antagonize canonical Wnt/β-catenin signaling by Frizzled family receptors and LRP5/LRP6 coreceptors.^3,56^ It has been demonstrated that DKK1 and DKK2 have important functions in endothelial function,^57–59^ including the role that DKK1 plays in accelerating proinflammatory response and atherosclerosis.^59^ In the present study, we found that the DKK1 level in human blood showed a significant positive association with atherosclerosis independently of DKK3 levels. In vitro study of cultured endothelial cells pretreated with DKK3 did induce changes in DKK1-induced interleukin-6 and monocyte chemoattractant protein-1 expression (online-only Data Supplement XII). It is important to note that LRP5 or LRP6 antagonist sclerostin and draxin did not inhibit DKK3-induced activation of the noncanonical Wnt signaling pathway (online-only Data Supplement XIII). Thus, it seems that DKK3 exerts its effect on endothelial functions related to atherosclerosis independently of other members of DKK family proteins.

In contrast with the other DKK family members, the specific receptors and relevant signaling pathways with which DKK3 interacts remain controversial. Several studies have demonstrated that DKK3 exerted its functions through the canonical Wnt/β-catenin signaling pathway by Kremen^20^ and LRP5.^60^ In contrast, other studies found no evidence that DKK3 binds to LRP5/6 or Krm1/2 or inhibits the canonical Wnt signaling pathway.^61,62^ In comparison with the canonical Wnt pathway, which is mainly involved in cell proliferation and differentiation, the noncanonical Wnt-planar cell polarity pathway interferes with cell adhesion, motility, and polarity.^38,63^ In the present study, we found that neither β-catenin expression nor its cellular location was changed in endothelial cells after DKK3 treatment. Instead, our data revealed that DKK3 induced cell migration through the activation of GTPase Rac1 but not RhoA and phosphorylation of JNK and c-jun. These results suggest that DKK3-induced cell migration is mediated by a ROR2-Dvl1-Rac1-JNK signaling pathway (online-only Data Supplement XIV).

In summary, in the current study, we have provided the first evidence that DKK3 potentially confers protection against atherosclerosis in human subjects and established that DKK3 affects atherosclerosis progression and neointimal formation in mouse models by influencing reendothelialization. Furthermore, we identified ROR2-Dvl1-Rac1-JNK as the potential signaling pathway that relays DKK3 signal in endothelial cells in vitro to induce cell migration. Taken together, the findings indicate that DKK3 could be an atheroprotective cytokine that might serve as a biomarker of endothelial integrity and repair and a potential therapeutic agent (eg, for improving both early stage reendothelialization and long-term outcome of patients postangioplasty). Although substantial knowledge on vascular risk factors has accumulated over the past years, insights into protective mechanisms are limited and require more extensive studies.

## Sources of Funding

This work was mainly supported by the British Heart Foundation (RG/14/6/31144), and partially by the Oak Foundation, National Natural Science Foundation of China (81370521, 81320157, and 81670400), The Importation and Development of High-Caliber Talents Project of Beijing Municipal Institutions (CIT&TCD20150325), The Key Science and Technology Project of Beijing Municipal Institutions (KZ201610025025), The Fok Ying-Tong Education Foundation (151041), and the Beijing Haiju Project. Drs Kiechl, Willeit, Mayr, and Weger were supported by the excellence initiative (Competence Centers for Excellent Technologies) of the Austrian Research Promotion Agency: Research Center of Excellence in Vascular Ageing, Tyrol (K-Project No. 843536) funded by the Wirtschaftsagentur Wien and Standortagentur Tirol.

## Disclosures

None.

## Supplementary Material
