# Supplementary material for: A Cytokine-Like Protein Dickkopf-Related Protein 3 Is Atheroprotective
Source: Circulation. 2017 Sep 11;136(11):1022–36. doi: 10.1161/CIRCULATIONAHA.117.027690 (PMC5598907; doi:10.1161/CIRCULATIONAHA.117.027690)
Supplement: Supplementary file 1 [file cir-136-1022-s001.pdf]

Expanded Methods

Study Population. Population recruitment was performed as part of the prospective community-based Bruneck Study[1, 2]. The survey area was located in the north of Italy (Bolzano Province). Special features of the study design and protocol have been described previously in detail[1-3]. At the study baseline in 1990, we recruited an age- and sex-stratified random sample from all inhabitants of Bruneck aged 40-79 years (125 women and 125 men each from the 5th to 8th decades of age, overall n=1000). A total of 919 subjects participated and had complete data assessment. Follow-up examinations were performed every five years with participation rates among survivors excceding 90%: n=826 in 1995, n=684 in 2000, and n=574 in 2005. The current study utilized data and blood samples collected in the 2000 and 2005 examinations. The appropriate ethics committees approved the study protocol and all study subjects gave their written informed consent before entering the study.

Clinical Examination and Laboratory Methods. Body mass index was calculated as weight divided by height squared (kg/m^2). Diabetes was diagnosed according to American Diabetes Association (ADA) criteria. Hypertension was defined as blood pressure ≥140/90 mm Hg (mean of three independent measurements obtained with a standard mercury sphygmomanometer after at least ten minutes of rest) or the use of anti-hypertensive drugs[1-5]. All other parameters were assessed by standard procedures as detailed prreviously[1-5]. Blood samples were collected from antecubital vein after subjects had fasted and abstained from smoking for ≥12 hours. Blood was collected in EDTA tubes and processed within 3 hours of collection. The APC-CFU assay and the APC culture assay were then performed promptly. Plasma was aliquoted and frozen for ELISA and other future use.

Enzyme-Linked Immunosorbent Assay (ELISA) for Plasma Soluble Molecules Measurements. The levels of DKK3 in human plasma were detected using R&D DKK3 ELISA kit (R&D, DY1118). The capture antibody was diluted to a working concentration without carrier protein. A 96-well ELISA microplate (R&D, DY990) was immediately coated with 100µl of capture antibody, sealed (sealer, R&D, DY992), and incubated overnight at 4 oC. The next day the solution was removed and the wells were washed 3 times with wash buffer (provided in the kit). Each well was then blocked with 300µl of blocking reagent (provided in the kit) and maintained at room temperature for at least 1 hour. The wells were washed with wash buffer 3 times. 100µl of samples (diluted 1:10-1:100) or standards were added. The plate was covered with an adhesive strip and incubated at room temperature for 2 hours. Each

well was washed 3 times with wash buffer. 100µl of detection antibody was added and the plate was covered again with an adhesive sealer and incubated at room temperature for 1 hour and then removed. 100µl of streptavidin-HRP (provided in the kit) was added per well followed by 3 washes with wash buffer and incubation at room temperature for 20 minutes (avoiding direct exposure to light). 100µl of substrate solution (provided in the kit) consisting of equal volumes of color reagent A (H2O2) and color reagent B (Tetramethylbenzidine) was added per well and incubated at room temperature for another 15-25 minutes. The reaction was stopped by adding in 50µl stop solution (2N sulphuric acid). The optical density of each well was determined immediately using a Tecan microplate reader set to 450 nm. The standard curve was created by plotting the concentration for each standard on the y-axis against the mean absorbance for each standard on the x-axis and drawing a best-fit curve through the points on the graph. The DKK3 concentration was calculated according to the standard curve. DKK1 levels were measured in serum with a commercial ELISA (Biomedica, Vienna, Austria): Intra- and inter-assay CVs were low at 3% each and the lower detection limit was 1.6 pmol/L.

The levels of plasma G-CSF, SDF1-α, VEGF and MMP-9 were also determined by using corresponding ELISA kits (Quantikine, R&D Systems, UK). All ELISA tests were carried out at room temperature on freshly thawed plasma samples. The concentrations of all cytokines were determined by comparison with a standard curve, following manufacturer's instruction. Other laboratory parameters were all examined by standard methods.

Angiogenic Promoting Cell (APC) Colony Forming Unit Assay (APC-CFU) and APC Culture Assay (APC number). Angiogenic promoting cell (APC) number and APC-CFU were assessed in 571 and 542 participants. Subjects with and without APCs number and APC-CFU did not differ in age, sex and expression of cardiovascular risk factors. Venous blood samples were collected as described above and the APC-CFU assay performed as described previously[6-8]. Briefly, peripheral blood mononuclear cells were isolated by density gradient centrifugation over lymphoprep (Axis-Shield, Oslo, Norway) at 800 G for 20 minutes. Peripheral blood mononuclear cells were resuspended in APC culture medium (M199 with 20%FCS and antibiotics) and then plated on fibronectin-coated 6-well plates at a concentration of 5 million cells per well. This step was designed to remove mature circulating endothelial cells which are rapidly adherent to fibronectin. Following culture for 48 hours, non-adherent cells were then aspirated, counted and, plated on fibronectin-coated 24-well plates at a concentration of 1 million cells per well. On day 5 of the assay, the medium was changed and on day 7 the endothelial colonies were counted manually. Strict guidelines were followed to ensure consistent counting of APC colonies. Two senior investigators who were blinded to the subjects' clinical status counted colonies. Reproducibility was

assessed over 50 samples comparing colony counts by the two individuals. The coefficient of variance was <10% in each case. The APCs in a minimum of two wells were counted and the average was then recorded. Reproducibility was assessed over 50 samples by comparing the APC numbers from the two individuals. The coefficient of variance was <10% in each case.

Scanning Protocol and Definition of Ultrasound End Points. The ultrasound protocol involves the scanning of the internal (bulbous and distal segments) and common carotid arteries (proximal and distal segments) of either side with a 10-MHz imaging probe[3, 9]. Atherosclerotic lesions were defined according to two ultrasound criteria: (1) wall surface (protrusion or roughness of the arterial boundary) and (2) wall texture (echogenicity). The maximum axial diameter of plaques (in millimeters) was assessed on the near and far walls at each of the eight vessel segments. for the follow-up between 2000 and 2005 two stages of atherosclerosis development/progression were differentiated: (1) early atherogenesis was defined as the occurrence of new plaques in previously normal vessel segments, and (2) advanced atherogenesis (incident stenosis) was assumed whenever the relative increase in the maximum plaque diameter exceeded twice the measurement error for the method and a lumen narrowing > 40% (diameter stenosis) was achieved. The two progression categories were highly reproducible [kappa coefficients > 0.8 (n=100)]. Further details on imaging procedures and measurement errors have been published elsewhere[3, 9, 10]. The intima-media thickness (IMT) was quantified at the far wall of plaque-free sections of the common carotid arteries as the distance between the lumen-intima and media-adventitia interface (intra-observer coefficient of variation, 7.9 percent (n=100))[3, 9].

Animals. All animal experiments were performed according to the protocols approved by the Institutional Committee for the Use and Care of Laboratory Animals. ApoE$^{-/-}$ mice were purchased from the Jackson Laboratory. For ApoE$^{-/-}$ mice genotyping, the following primers were employed: oIMR180 (5'-GCC TAG CCG AGG GAG AGC CG-3'), oIMR181 (5'-TGT GAC TTG GGA GCT CTG CAG C-3'), and oIMR182 (5'-GCC GCC CCG ACT GCA TCT-3'). DKK3$^{-/-}$ mice were generated as described previously[11]. Three genotypes of DKK3$^{-/-}$, DKK3$^{-/+}$ and DKK3$^{+/+}$ mice were identified using PCR (primers:5-GATAGCTTTCCGGGACACAC-3; 5-TCCATCAGCTCCTCCA CCTCT-3; 5-TAAGTTGGGTAACGCCAGGGT-3). ApoE$^{-/-}$ mice were crossed with DKK3$^{-/-}$ mice in our laboratory, and heterozygous offspring were mated to produce ApoE$^{-/-}$ mice lacking DKK3 (DKK3$^{-/-}$ApoE$^{-/-}$). The mice were maintained on a light/dark (12/12 h) cycle at 22°C and received food and water ad libitum. The genetic background of all mice used in the present study was C57BL/6.

Creation of Chimeric Mice. The procedure used for creating chimeric mice was similar to previously described[12]. In brief, bone marrow transplantation was carried out on the DKK3$^{+/+}$ mice and DKK3$^{-/-}$ mice separately. Bone marrow cells were obtained from the femurs and tibias of either DKK3$^{+/+}$ or DKK3$^{-/-}$ mice (donors) and injected ($1x10^{7}$cells in 0.2ml) into the tail veins of the 6-8 week old DKK3$^{-/-}$ mice or DKK3$^{+/+}$ mice (recipients), which received lethal irradiation (950 Rads) before. The measurement of DKK3 level in peripheral blood was performed 3 weeks after bone marrow transplantation.

Tissue Harvesting and Lesion Analysis. Mice were anesthetized by intraperitoneal injection of pentobarbital atrium (50mg/kg b.w.). Blood was obtained from inferior vena cava for lipid analysis. The heart was harvested intact and stored immediately in liquid nitrogen and the whole length of the aorta was stored in formalin at 4°C. 8-μm thick frozen sections were obtained from the heart and were stained with Oil Red O as described elsewhere[13]. Aortas were opened longitudinally and fixed on a silicon bed with stainless steel pins (Fine Science Tool, USA) with the intima exposed. Oil Red O staining was performed. Lesion areas were measured and quantified using a computer software AxioVision™ as described previously.

Lipid Measurement. Blood from the mice was collected, centrifuged and the serum was kept for analysis. Serum cholesterol and triglyceride were measured with the Infinity™ Kit (Sigma) for cholesterol and triglyceride respectively.

Endothelial Damage Assays *in vivo.* Evans blue dye stains blue in the areas where endothelium is damaged or dysfunctional[14]. Mice received intravenous Evans blue dye injection, and their aortas harvested, opened and blue areas were measured using a computer software AxioVision™. For scanning electron microscope analysis, mice were killed, and the aortas were fixed *in situ* with 2% formaldehyde plus 2% glutaformaldehyde and harvested. Aortic samples were fixed and processed as described previously. The samples were observed using a scanning electron microscopy in our central facility laboratory.

Mouse Femoral Artery Denudation Injury. The mice were anesthetized and the surgical procedure was similar to that described previously[15, 16]. Removal of the endothelium of the common femoral arteries was achieved by 3 passages of a 0.25 mm angioplasty guidewire (Advanced Cardiovascular Systems). After removal of the wire, the branch of the femoral artery was ligated, and the wound was closed.

Morphometric Analysis and Quantification of Lesion Formation. The femoral arteries were harvested at different times after the operation. The specimens were fixed in 4% formaldehyde for H&E staining. Sections (5μm)

were collected at 100µm intervals (10 sections per fragment), mounted on slides, and stained with H&E for morphometric analysis. The procedure used for lesion quantification was similar to that described previously[17]. Briefly, using Axiovision software, the area of neointima in a given image was highlighted and quantified (mm^2) automatically. 10 sections were analyzed per vessel sample. Meanwhile, the thickness of the neointima and media of the vessel was examined. Because native media thickness is variable (reflecting the diameter of the artery), neointima thickness was assessed in the ratio of intima to intima plus media thickness. Re-endothelialization was defined by endothelial coverage of the luminal surface and assessed by x400 microscopic examination.

***En Face* Staining.** The mice were sacrificed after 48 hours, their aortas were harvested, processed and fixed as described previously[18] and probed for 1 hour at room temperature with rat anti-mouse antibodies against eNOS (BD Biosciences; 1:20 dilution). The secondary antibody used was rabbit anti-rat conjugated with Cy3 (DAKO Corp; 1:50 dilution).

***In vivo* Matrigel Plug Assay.** DKK3 or BSA (50 µl, 100µg/ml) were mixed well with and 250 µl Matrigel on ice, and injected subcutaneously into the mice. 6 injections were conducted for each group. The plugs were harvested 14 days later. Samples were fixed in liquid nitrogen, cryosections were prepared and immunofluorescence staining was then performed.

Immunofluorescence Staining. Frozen or paraffin sections on HUVECs were fixed with 4% paraformaldehyde, permeabilized with 0.1% Triton X-100 (Sigma) and blocked with 5% normal donkey serum (Dako) for 1 hour at room temperature. Incubation of tissue sections or cells with primary antibodies was performed at 4^oC overnight, followed by incubation with secondary antibodies (Corresponding fluorescent-conjugated IgG antibodies were used as secondary antibodies (Invitrogen)) for 45 mins at 37^oC. Then tissue sections or cells were counterstained with DAPI (Sigma) for 5 mins at room temperature and mounted in fluorescent mounting media (Dako). Images were acquired using an Olympus IX81 microscope and assessed using Volocity software (PerkinElmer) or a Leica SP5 confocal microscope and LAS AF lite software. Total positive cells were counted using images obtained from the microscope, and these images were also used for calculating the percentage of positive cells.

Isolation and Culture of Cells. *HUVEC.* Human umbilical vein endothelial cells (HUVECs) were purchased from Lonza (C2519A pooled donor) and cultured in complete Endothelial Growth Medium, EGM-2 Bullet kit (Lonza, CC3162). The cells were placed in a gelatin-coated plastic flask, incubated at

37℃，5% CO_2 and were passaged every other day at a 1 to 3 ratio. The experiments were carried on HUVECs of passage 2-5.

MLEC. Mouse lung endothelial cells (MLEC) were isolated using collagenase/dispase digestion method as previously described. Briefly, 8-10 days old pups of C57BL/6J mice were euthanized before excision of three lung lobes of each mouse aseptically. The tissue was minced finely followed by enzymatic digestion with collagenase/dispase for 45 minutes at 37°C on a rotator. A single cell suspension was obtained by triturating tissue clumps and passing through cell strain before collecting cell pellets by centrifugation at 400g for 5 minutes. Then the cells were resuspended and sorted with anti-PECAM-1 immunomagnetic microbeads to obtain MLEC. According to the instruction of magnetic cell sorting kits (MACS) (Miltenyi Biotec, GmbH, Bergisch Gladbach, Germany), briefly, the cells were incubated with the PECAM-1-conjugated/coated microbeads at 4°C for 15 minutes and then sorted using MACS columns (Miltenyi Biotec). The PECAM-1 positive cells were plated on a gelatin-coated T75 flask and changed EGM-2 medium 4 hours later to remove non-adherent cells and platelets.

Mouse Peritoneal Macrophages. The isolation of mouse peritoneal macrophages was described as in previous studies[14]. Briefly, C57BL/6J mice were received an intraperitoneal (IP) injection of either 0.9% saline, 3% thioglycollate medium (Sigma-Aldrich) or recombinant mouse DKK3 protein (Thermo Fisher Scientific) in each mouse (1μg/20g). After 3 days, all the mice in different groups were euthanized by cervical dislocation, and 10 ml of cold sterile 3% Fetal Bovine Serum (FBS) PBS was injected through the peritoneal wall into each mouse. After injection, the peritoneal fluid was then withdrawn and centrifuged at 200g for 10 minutes at 4°C. The supernatant was discarded and cell components were analyzed by FACs analysis.

CHO-K1 Cells. Chinese hamster ovary, CHO-K1 cell line was purchased from ATCC (CCL-61), and cultured in Nutrient Mixture F-12 Ham medium (Sigma N4888) supplemented with 2mM Glutamine and 10% Fetal Bovine Serum (FBS). The cells were passaged every other day at a 1 to 6 ratio.

FACS Analysis. Mouse peritoneal macrophages were blocked with CD16/CD32 (Mouse BD Fc Block™) (BD Biosciences) for 5 minutes on ice before addition of antibodies CD11b, CD3, B220 or their corresponding IgG control for 30 minutes on ice before analysis by FACS in order to test the populations of macrophage, T cell and B cell in isolated peritoneal macrophages after stimulation with saline, thioglycollate or DKK3 recombinant protein in different mice. Data analysis was carried out using FlowJo software.

Antibodies and Inhibitors. Primary antibodies were goat anti-DKK3 (R&D Systems, AF1118), rabbit anti-HA tag antibody (ab9110), rabbit anti-Phospho-SAPK/JNK (Thr183/Tyr185) (cell signaling 4668), rabbit anti-SAPK/JNK (cell signaling 9252), rabbit anti-Phospho-c-jun (Ser63/73) (SC16312-R), rabbit anti-c-jun (sc-1694), rabbit anti-FAK (phosphor Y397) antibody (ab4803), rabbit anti-paxillin (ab32084) and rabbit anti-ROR2 (ab92379), rabbit Anti-Dishevelled/Dvl1 antibody (ab106844), mouse anti-Rac1 (Millipore 05-389), mouse anti-RhoA (Cytoskeleton ARH04), mouse anti-active-β-catenin (Millipore 05-665), rabbit anti-β-catenin (ab32572), mouse anti-α-tubulin (Sigma T9026). Conjugated flow cytometry antibodies FITC anti-CD11b antibody (101205), FITC rat IgG2b, κ Isotype ctrl antibody (400605), PE anti-CD3 antibody (100205), PE rat IgG2b, κ Isotype ctrl antibody (400607) and APC anti-CD45R/B220 antibody (103211), APC rat IgG2a, κ Isotype ctrl antibody (400511) were purchased from Biolegend. Secondary antibodies for immunostaining were anti-mouse Alexa Fluor 546, anti-rabbit Alexa Fluor 546 and anti-rat Alexa546 and were purchased from Invitrogen. Human DKK1 (5439-DR-010), Draxin (6148-DR-025) and SOST/Sclerostin (1406-ST-025) were purchased from R&D System. Cells were also counterstained with Alexa Fluor® 488 Phalloidin (Life technologies A12379) and 10μg/ml 4', 6-diamidino-2-phenylindole (DAPI). Secondary antibodies for Western Blotting were purchased from Dako. JNK inhibitor SP600125 ($C_{14}H_8N_2O$) was purchased from Calbiochem.

Plasmids and Cell Transfection. pcDNA3-EGFP-Rac1-Q61L (plasmid 12981), pcDNA3-EGFP-Rac1-T17N (plasmid 12982), pcDNA3-EGFP-RhoA-Q63L (plasmid 12968), pcDNA3-EGFP-RhoA-T19N (plasmid 12967) were purchased from Addgene. HUVECs were transfected with above plasmids using Lipofectamine 3000 (Thermo Fisher) in Opti-MEM™ (Thermo Fisher) medium according to the manufacturer's instructions. After incubation at 37°C for 6 hours, the medium was changed to complete EGM-2 medium and migration assays were performed on transfected HUVECs 24 hours post transfection.

Adenovirus and Cell Transfection. Human DKK3-HA adenovirus (Abm 085672A) and Adeno CMV-null (Abm 000047A) were purchased from Abm and amplified in HEK293 cells as described previously1. The empty vector virus (Adeno CMV-null) was applied as a negative control in experiments. For overexpression of DKK3 in either CHO cells supernatant or HUVECs, 70% confluent CHO cells or HUVECs were infected with Adeno-DKK3-HA or Adeno-CMV null at a multiplicity of infection (MOI) of 10 for 6 hours before replacement of fresh serum free F-12 basal medium. The supernatant from CHO cells was harvested 48 hours after transduction and in which DKK3 concentration was determined by human DKK3 DuoSet ELISA kit (R&D

DY1118). The evaluations of DKK3 overexpression in HUVECs were performed 48-72 hours after transfection.

BrdU Proliferation Assay. HUVECs were treated with/without human DKK3 recombinant protein in 0.1% FBS EBM-2 medium or Adeno-DKK3-HA/Adeno-CMV null transfected CHO cells supernatant at indicated time points on a gelatine coated 96-well culture plate. The proliferation assay was performed using a Cell Proliferation ELISA, BrdU (colorimetric) (Roche). According to the manufacturer's instructions, the BrdU labeling solution was incubated with cells for 2 hours at 37℃ prior to fixation of cells. Then the fixed cells were incubated with BrdU peroxidase-conjugated antibody for 90 minutes at room temperature followed by incubation of substrate solution and stop solution until the color development was sufficient to be detected. The absorbance was measured at 450nm with correction at 690nm. The proliferation of HUVECs was expressed as the fold of mean values of absorbance compared to their corresponding controls.

Annexin V Apoptosis Assay. Apoptosis assay was performed on HUVECs using FITC Annexin V Apoptosis Detection Kit (BD Pharmingen^{TM}) after treatment with/without human DKK3 recombinant protein in 0.1% FBS EBM-2 medium or Adeno-DKK3-HA/Adeno-CMV null transfected CHO cells supernatant at indicated time points. As directed by manufacture's instruction, $1x10^5$ cells of each sample were incubated with FITC Annexin V and Propidium Iodide (PI) together for 15 minutes at room temperature protected from light. Unstained cells, cells stained with FITC Annexin V or PI only severed as controls. The apoptotic cells were identified by FACS immediately after staining.

Transwell Chemotaxis Assay. Migration chemotaxis assay was performed by applying 24-well Boyden chambers with 8 μm pore size polycarbonate membranes (Corning) as described previously[19]. HUVECs were seeded onto the upper chamber at $1x10^5$ cells in 0.1% FBS EBM-2 basal medium, while the bottom chamber contained either 0.1% FBS EBM-2 basal medium with indicated concentrations of recombinant human DKK3 or Adeno-DKK3-HA/Adeno-CMV null overexpressed CHO cells supernatant. 0.1% FBS EBM-2 basal medium served as negative control for the comparison with recombinant human DKK3. After incubation for 6 hours at 37°C, the cells remained on the upper side of the filters were removed by a cotton swab. The migrated cells on the underside of the membrane were fixed with 4% paraformaldehyde prior to staining with 0.1% crystal violet solution for 15 minutes. Data was expressed as the fold of migrated HUVECs compared to their corresponding controls.

Scratch-Wound Assay. In each well of a 24-well plate, a straight scratch was made by a pipette tip to stimulate a 'wound' through the middle of 100% confluent HUVECs monolayer. The cells were treated with 0.1% FBS EBM-2 basal medium with/without indicated concentrations of recombinant human DKK3 or Adeno-DKK3-HA/Adeno-CMV null overexpressed CHO cells supernatant. After 6 hours incubation, the migration of HUVECs into the "wound" area was quantified as the mean number of the migrated cells.

RT-PCR and Quantitative Real Time Polymerase Chain Reaction (qPCR). Total RNA was isolated from HUVECs using a QIAGEN RNeasy Mini kit according to the manufacturer's instructions. 1 μg RNA was reverse transcribed into cDNA using QuantiTect® Reverse Transcription Kit (Qiagen) in a 20 μl reaction. Then the qPCR was performed using 20ng of cDNA per sample with a SYBR Green Master Mix in a 20 μl reaction. Ct values were measured using the Eppendorf Mastercycler ep Realplex and GAPDH was used as an endogenous control to normalize the amounts of RNA in each sample. The primer sets are:

ROR2, Forward Primer 5'-GTGCGGTGGCTAAAGAATGAT-3'
Reverse Primer 5'-ATTCGCAGTCGTGAACCATATT-3'
DVL1, Forward Primer 5'-GAGGGTGCTCACTCGGATG-3'
Reverse Primer 5'-GTGCCTGTCTCGTTGTCCA-3'
DKK3, Forward 5'-AGGACACGCAGCACAAATTG-3'
Reverse 5'-CCAGTCTGGTTGTTGGTTATCTT-3'
IL-6, Forward 5′-TTG CCT TCT TGG GAC TGA TGC T-3′
Reverse 5′-GTA TCT CTC TGA AGG ACT CTG G-3′
MCP-1, Forward 5'-AGTAGGCTGGAGAGCTACAA-3'
Reverse 5'-GTATGTCTGGACCCATTCCTTC-3'

Western Blot Analysis. Harvested cells were lysed with RIPA lysis buffer (Thermo Fisher) plus cOmplete Protease Inhibitor Cocktail Tablets (Roche), PhosSTOP Phosphatase Inhibitor Cocktail Tablets (Roche) and proteins were sequentially measured using DC™ Protein Assay (Bio-Rad). 40 μg of total protein lysate for each sample was loaded into each well of 4-12% Bis-Tris Protein gels (NuPAPE, Novex) before being transferred to an Immobilon®-FL transfer membrane (Millipore), followed by a standard western blotting procedure.

Rac1, RhoA GTPase Activation Assay. The activation assay of GTP-bound Rac1 or GTP-bound RhoA were carried out according to the manufacturer's instructions of Rac1 Pull-down Activation Assay Kit (Cytoskeleton) and RhoA Pull-down Activation Assay Kit (Cytoskeleton) respectively. Briefly, cells were stimulated with the indicated treatments then harvested within cell lysis buffer

supplemented with protease inhibitor cocktail (reagents all supplied by kits). Then Rac1 and RhoA activity were assessed using GST-tagged p21 binding domain (PBD) of PAK agarose beads or glutathione S transferase (GST)-tagged Rho-binding domain (RBD) of Rhotekin beads to pull-down GTP-Rac1 or GTP-RhoA from lysates separately. Aliquots were collected for total Rac1 or Cdc42 (input) and GTPγS (positive) and GDP (negative) control analysis. Samples were separated by 4–12% Bis-Tris gels, transferred to PVDF membranes, and blotted with Rac1 (Millipore, 05-389) or RhoA antibody (Cytoskeleton, ARH03).

Statistical Analysis

Population study. The data were analyzed using the SPSS 24 software package. Levels of variables according to DKK3 tertile groups were presented as mean values ± SD or as medians with corresponding 25th and 75th percentiles (continuous variables), and percentages (dichotomous variables). Associations between DKK3 level (predictor variable) and vascular risk factors, life style and demographic variables, IMT and atherosclerosis progression were assessed using linear and logistic regression analysis. Levels of variables with a markedly skewed distribution were $\log_e$-transformed to satisfy the assumption of normality and constant variance of the residuals. The multivariate models focusing on IMT or atherosclerosis progression included the following covariates: age (years), sex (female, male), smoking (cigarettes/day), hypertension, HDL and LDL cholesterol, triglycerides, hsCRP, creatinine, body mass index, wait-to-hip ratio, chronic infections, fasting glucose, and physical activity (sports score). A two-sided p value < 0.05 was considered significant.

In vivo and in vitro studies. Data for *in vivo* and *in vitro* studies are presented as the mean ± standard error of the mean (S.E.M.) of at least three separate experiments. The analysis was performed using Graphpad Prism V.6 (GraphPad Software, San Diego CA) using t-test between two groups and analysis of variance (one-way ANOVA) followed by Dunnett's multiple comparison test for more than two groups. A p value<0.05 was considered significant.

References

1. Willeit J, Kiechl S. Prevalence and risk factors of asymptomatic extracranial carotid artery atherosclerosis. A population-based study. *Arterioscler Thromb.* 1993;13:661-668.
2. Xu Q, Willeit J, Marosi M, Kleindienst R, Oberhollenzer F, Kiechl S, Stulnig T, Luef G, Wick G. Association of serum antibodies to heat-shock protein 65 with carotid atherosclerosis. *Lancet.* 1993;341:255-259.

3. Kiechl S, Willeit J. The natural course of atherosclerosis. Part II: vascular remodeling. Bruneck Study Group. *Arterioscler Thromb Vasc Biol.* 1999;19:1491-1498.
4. Xu Q, Schett G, Perschinka H, Mayr M, Egger G, Oberhollenzer F, Willeit J, Kiechl S, Wick G. Serum soluble heat shock protein 60 is elevated in subjects with atherosclerosis in a general population. *Circulation.* 2000;102:14-20.
5. Kiechl S, Egger G, Mayr M, Wiedermann CJ, Bonora E, Oberhollenzer F, Muggeo M, Xu Q, Wick G, Poewe W, Willeit J. Chronic infections and the risk of carotid atherosclerosis: prospective results from a large population study. *Circulation.* 2001;103:1064-1070.
6. Grisar J, Aletaha D, Steiner CW, Kapral T, Steiner S, Seidinger D, Weigel G, Schwarzinger I, Wolozcszuk W, Steiner G, Smolen JS. Depletion of endothelial progenitor cells in the peripheral blood of patients with rheumatoid arthritis. *Circulation.* 2005;111:204-211.
7. Hill JM, Zalos G, Halcox JP, Schenke WH, Waclawiw MA, Quyyumi AA, Finkel T. Circulating endothelial progenitor cells, vascular function, and cardiovascular risk. *N Engl J Med.* 2003;348:593-600.
8. George J, Herz I, Goldstein E, Abashidze S, Deutch V, Finkelstein A, Michowitz Y, Miller H, Keren G. Number and adhesive properties of circulating endothelial progenitor cells in patients with in-stent restenosis. *Arterioscler Thromb Vasc Biol.* 2003;23:e57-60.
9. Kiechl S, Willeit J. The natural course of atherosclerosis. Part I: incidence and progression. *Arterioscler Thromb Vasc Biol.* 1999;19:1484-1490.
10. Willeit J, Kiechl S, Oberhollenzer F, Rungger G, Egger G, Bonora E, Mitterer M, Muggeo M. Distinct risk profiles of early and advanced atherosclerosis: prospective results from the Bruneck Study. *Arterioscler Thromb Vasc Biol.* 2000;20:529-537.
11. Barrantes Idel B, Montero-Pedrazuela A, Guadano-Ferraz A, et al. Generation and characterization of dickkopf3 mutant mice. *Mol Cell Biol.* 2006;26:2317-2326.
12. Hu Y, Zhang Z, Torsney E, Afzal AR, Davison F, Metzler B, Xu Q. Abundant progenitor cells in the adventitia contribute to atherosclerosis of vein grafts in ApoE-deficient mice. *J Clin Invest.* 2004;113:1258-1265.
13. Hu Y, Davison F, Zhang Z, Xu Q. Endothelial replacement and angiogenesis in arteriosclerotic lesions of allografts are contributed by circulating progenitor cells. *Circulation.* 2003;108:3122-3127.
14. Foteinos G, Afzal AR, Mandal K, Jahangiri M, Xu Q. Anti-heat shock protein 60 autoantibodies induce atherosclerosis in apolipoprotein E-deficient mice via endothelial damage. *Circulation.* 2005;112:1206-1213.
15. Roque M, Fallon JT, Badimon JJ, Zhang WX, Taubman MB, Reis ED. Mouse model of femoral artery denudation injury associated with the rapid accumulation of adhesion molecules on the luminal surface and recruitment of neutrophils. *Arterioscler Thromb Vasc Biol.* 2000;20:335-342.
16. Lindner V, Fingerle J, Reidy MA. Mouse model of arterial injury. *Circ Res.* 1993;73:792-796.

17. Torsney E, Mayr U, Zou Y, Thompson WD, Hu Y, Xu Q. Thrombosis and neointima formation in vein grafts are inhibited by locally applied aspirin through endothelial protection. *Circ Res.* 2004;94:1466-1473.
18. Zeng L, Xiao Q, Chen M, et al. Vascular endothelial cell growth-activated XBP1 splicing in endothelial cells is crucial for angiogenesis. *Circulation.* 2013;127:1712-1722.
19. Wong MM, Chen Y, Margariti A, Winkler B, Campagnolo P, Potter C, Hu Y, Xu Q. Macrophages Control Vascular Stem/Progenitor Cell Plasticity Through Tumor Necrosis Factor-alpha-Mediated Nuclear Factor-kappaB Activation. *Arterioscler Thromb Vasc Biol.* 2014;34:635-643.

Table S1. Association of DKK3 level with common carotid artery IMT and progression of carotid atherosclerosis.

Incident atherosclerosis (2000 to 2005)

	OR (95% CI) for a 1-SD higher DKK3 level	P value
Model 1	0.64 (0.42-0.97)	0.035
Model 2	0.61 (0.39-0.95)	0.030

Incident stenosis (2000 to 2005)

	OR (95% CI) for a 1-SD higher DKK3 level	P value
Model 1	0.56 (0.38 to 0.83)	0.004
Model 2	0.53 (0.35 to 0.82)	0.004

Intima-media thickness IMT (mm)

	Regression coefficient (95% CI) for a 1-SD unit higher DKK3 level	P value
Model 1	-0.014 (-0.027 to -0.002)	0.020
Model 2	-0.012 (-0.024 to 0.001)	0.065

ORs (odds ratios) were derived from logistic regression analysis of incident carotid atherosclerosis/stenosis on baseline (2000) DKK3 levels, age and sex (Model 1) plus smoking (cigarettes/day), hypertension, HDL and LDL cholesterol, $\log_e$-transformed triglycerides, $\log_e$-transformed hsCRP, creatinine, body mass index, wait-to-hip ratio, chronic infections, fasting glucose and physical activity (sports score) (Model 2). The analysis on incident plaques focused on subjects free of carotid atherosclerosis in 2000 (n=259, incident plaques were observed in 59 individuals) and that on incident stenosis considered subjects with pre-existent carotid atherosclerosis only (n=332, incident stenosis was observed in 63 individuals). Regression coefficients were derived from linear regression models focusing on the common carotid artery intima-media thickness (IMT) measured in 2000 (same adjustment).
The SD of DKK3 level is 19.11.

Table S2. Levels of cytokines and APCs according to DKK3 tertile groups in the Bruneck Study.

Variable	DKK3 Tertile I (<65 ng/mL)	DKK3 Tertile II (65-74 ng/ml)	DKK3 Tertile III (≥75 ng/ml)	P value*	P value†
APC-CFUs (per 10^6 PBMNC)	104 (0-222)	83 (1-294)	79 (0-314)	0.900	0.633
APC number (per 10^6 PBMNC)	611 (285-998)	524 (192-1104)	489 (162-1086)	0.148	0.485
G-CSF (pg/ml)	8.5 (4.1-15.7)	10.1 (4.3-16.6)	10.0 (4.3-18.3)	0.120	0.511
MMP9 (ng/ml)	59.0 (34.8-110.0)	67.9 (38.9-102.0)	67.0 (31.7-121.7)	0.530	0.411
VEGF (pg/ml)	62.8 (27.3-149.2)	91.9 (32.0-186.5)	66.3 (28.0-147.5)	0.731	0.839
SDF-1 (pg/ml)	2465 (2247-2703)	2467 (2220-2804)	2693 (2362-3010)	<0.001	0.021
sRANKL (pmol/l)	1.2±0.9	1.3±1.2	1.2±1.1	0.701	0.356
Osteoprotegerin (pmol/L)	3.7±1.0	4.1±1.8	4.4±1.5	<0.001	0.130

P values were derived from linear regression analyses (continuous variables) and logistic regression analyses (categorical variables) comparing levels of study characteristics (outcome) across DKK3 tertile groups (predictor). Analyses were either unadjusted (*) or adjusted for age and sex (†).

Angiogenic promoting cell (APC) number and colony forming units (APC-CFUs) were available in 477 and 451 individuals, respectively. Cytokines levels were available as follows: granulocyte colony-stimulating factor (G-CSF) n=218, metalloproteinase 9 (MMP9) n=468, vascular endothelial growth factor (VEGF) n=311, and stromal cell-derived factor 1 (SDF-1) n=477.

Figure S1 **Yu et al.**

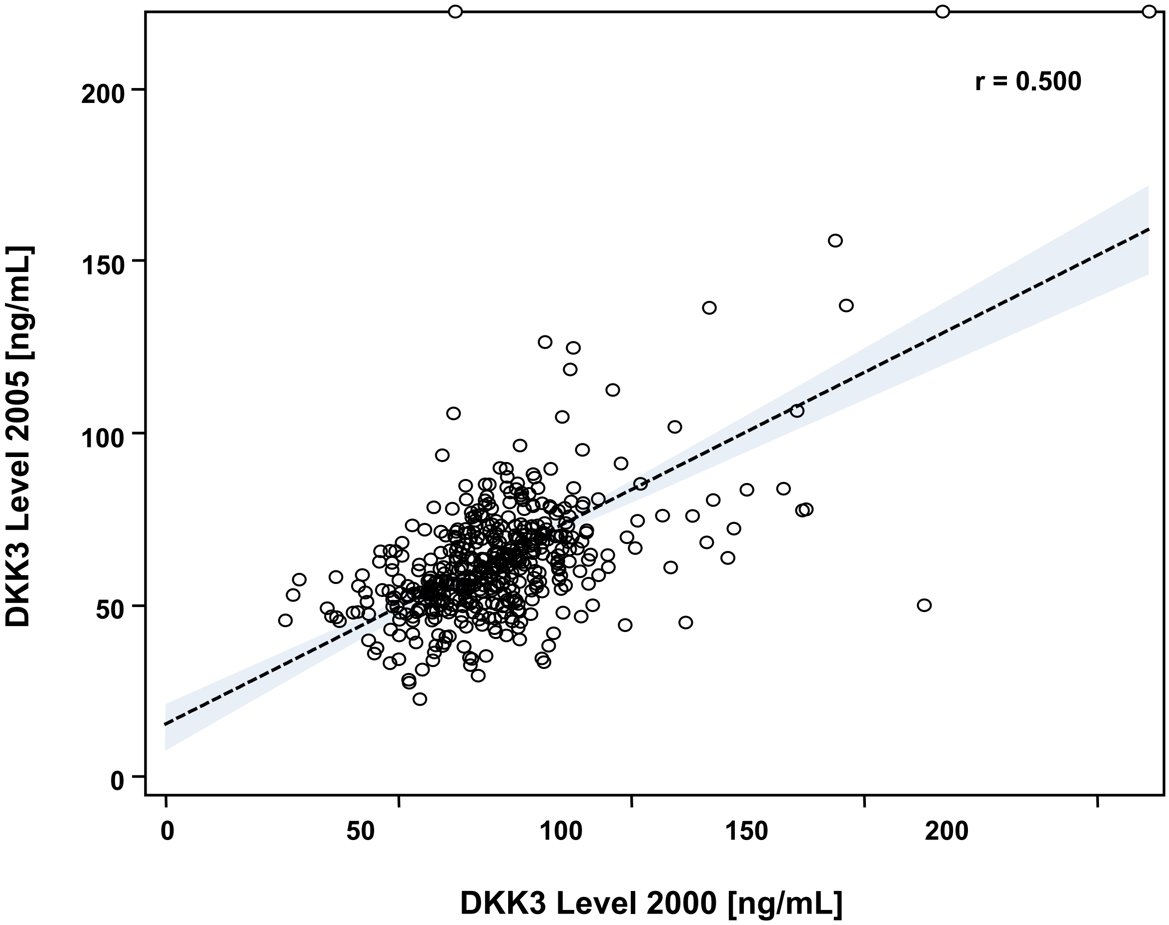

Figure S1. DKK3 levels of each human subject in year 2000 and 2005. Scatterplot of DKK3 levels measured in 2000 and 2005 in the same individuals with regression line and 95% CI. The open circles on the upper border of the figure indicate individuals with DKK3 levels higher than the range of the y-axis.
r, Spearman's rank correlation coefficient.

Figure S2 Yu et al.

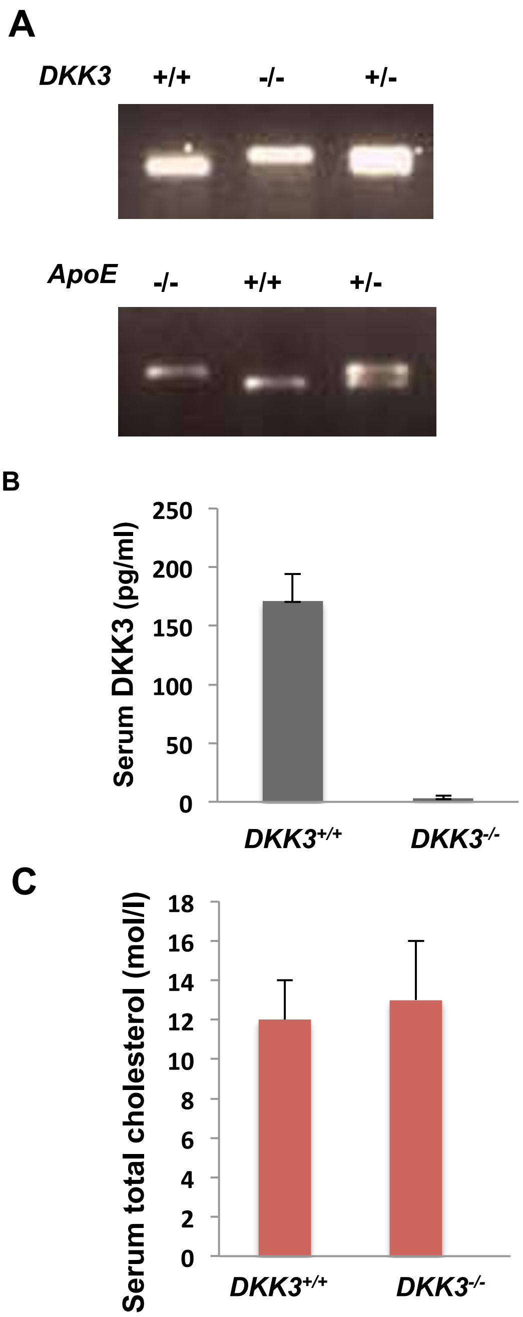

Figure S2. Characterization of *DKK3*$^{-/-}$*/ApoE*$^{-/-}$ mice. **A**) Phenotyping of *DKK3* and *ApoE* double knockout mice as identified by RT-PCR. **B**) ELISA measurement of serum DKK3 levels in DKK3 and ApoE double knockout mice. Note that DKK3 is almost undetectable in *DKK3*$^{-/-}$ *ApoE*$^{-/-}$ mice. **C**) Serum total cholesterol of *DKK3*$^{-/-}$*/ApoE*$^{-/-}$ and *DKK3*$^{+/+}$*/ApoE*$^{-/-}$ mice with 20 weeks of age was measured with a kit.

Figure S3 **Yu et al.**

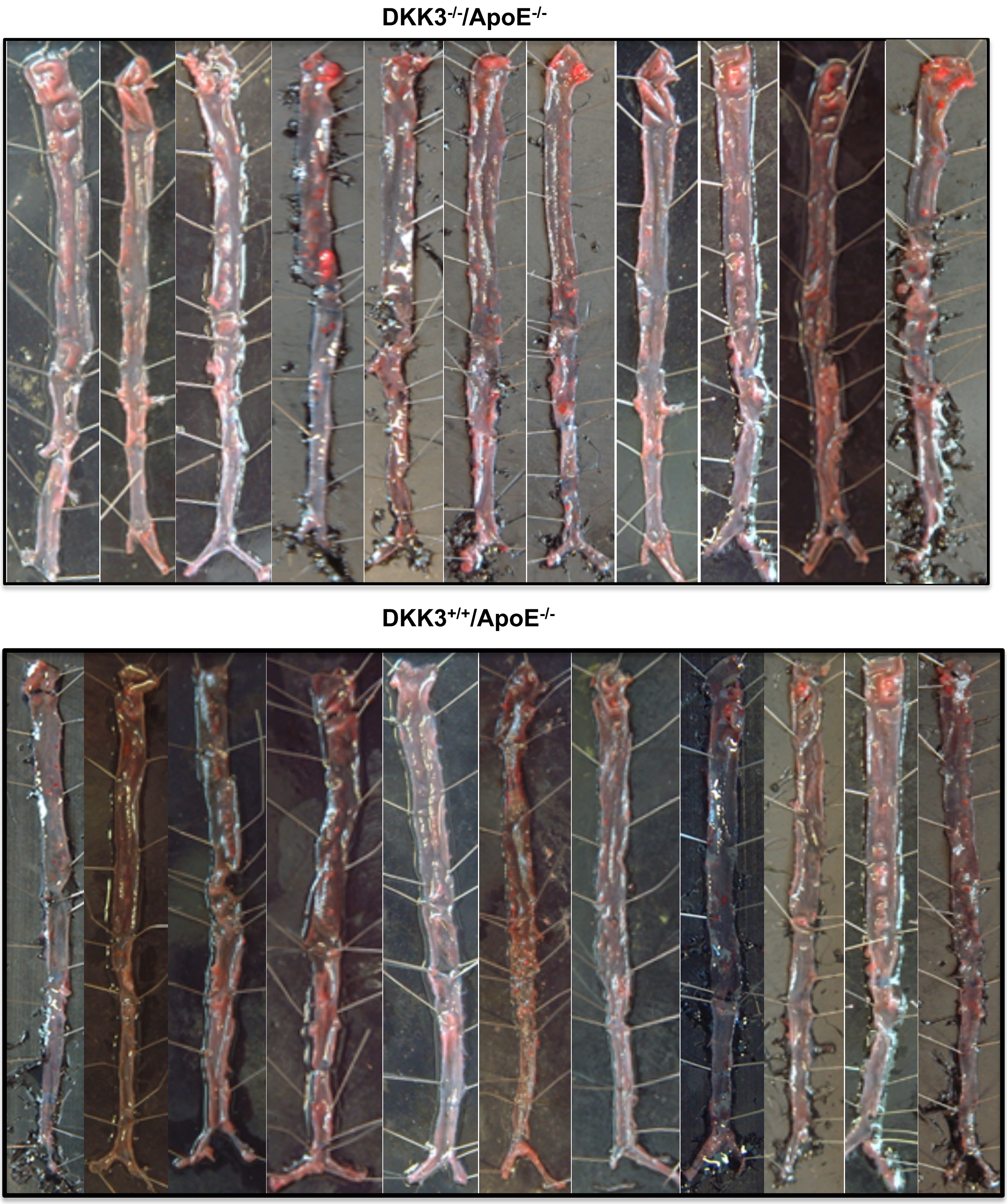

Figure S3. Atherosclerotic lesions in *DKK3$^{-/-}$/ApoE$^{-/-}$* mice. Mice receiving normal chow diet were killed at 20 weeks, and aortas were harvested, opended, mounted and stained with oil Red O.

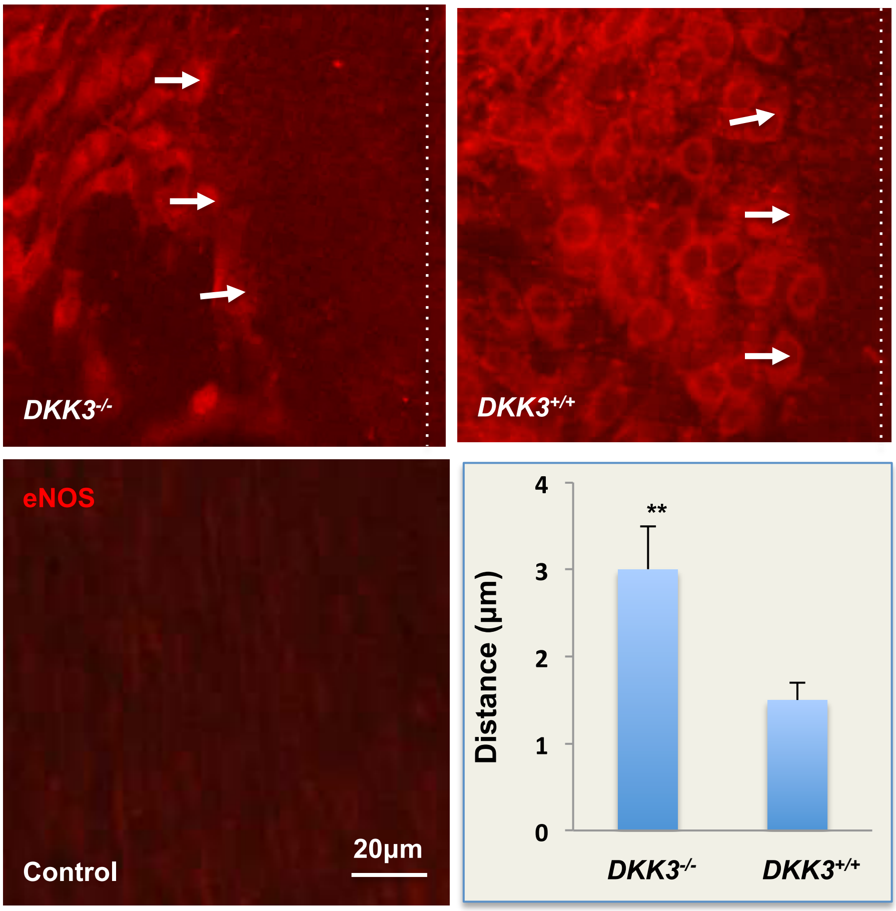

Figure S4. Delayed reendothelialization in injured vessels in *DKK3*$^{-/-}$ *ApoE*$^{-/-}$ mice. To establish reendothelialization model, femoral arteries of mice were injured. *En face* preparation of the vessel was stained for endothelial marker eNOS 1 week after wire injury. Dotted lines in images indicate the middle of the injured vessel, and the arrows indicate the direction of cell migration. Quantitative data of endothelial migration were presented in graphic data (n=6), **p<0.01, significant difference between two groups.

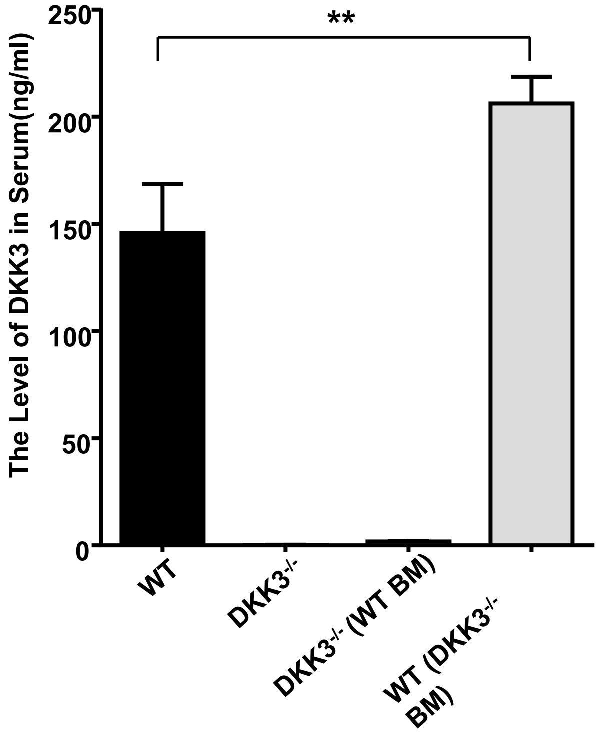

Figure S5. The source of DKK3 *in vivo*. A. Using chimeric mice model, 2 weeks after bone marrow transplantation, the level of DKK3 in peripheral blood was measured using a murine DKK3 Quantikine ELISA kit. The quantification in the graph shown mean ± SEM, n=5/group. **p<0.01. Wild-type (WT) to DKK3$^{-/-}$ bone marrow transplantation, wild type mice with DKK3$^{-/-}$ bone marrow. DKK3$^{-/-}$ WT bone marrow transplantation, DKK3$^{-/-}$ mice with wild-type bone marrow.

Figure S6 **Yu et al.**

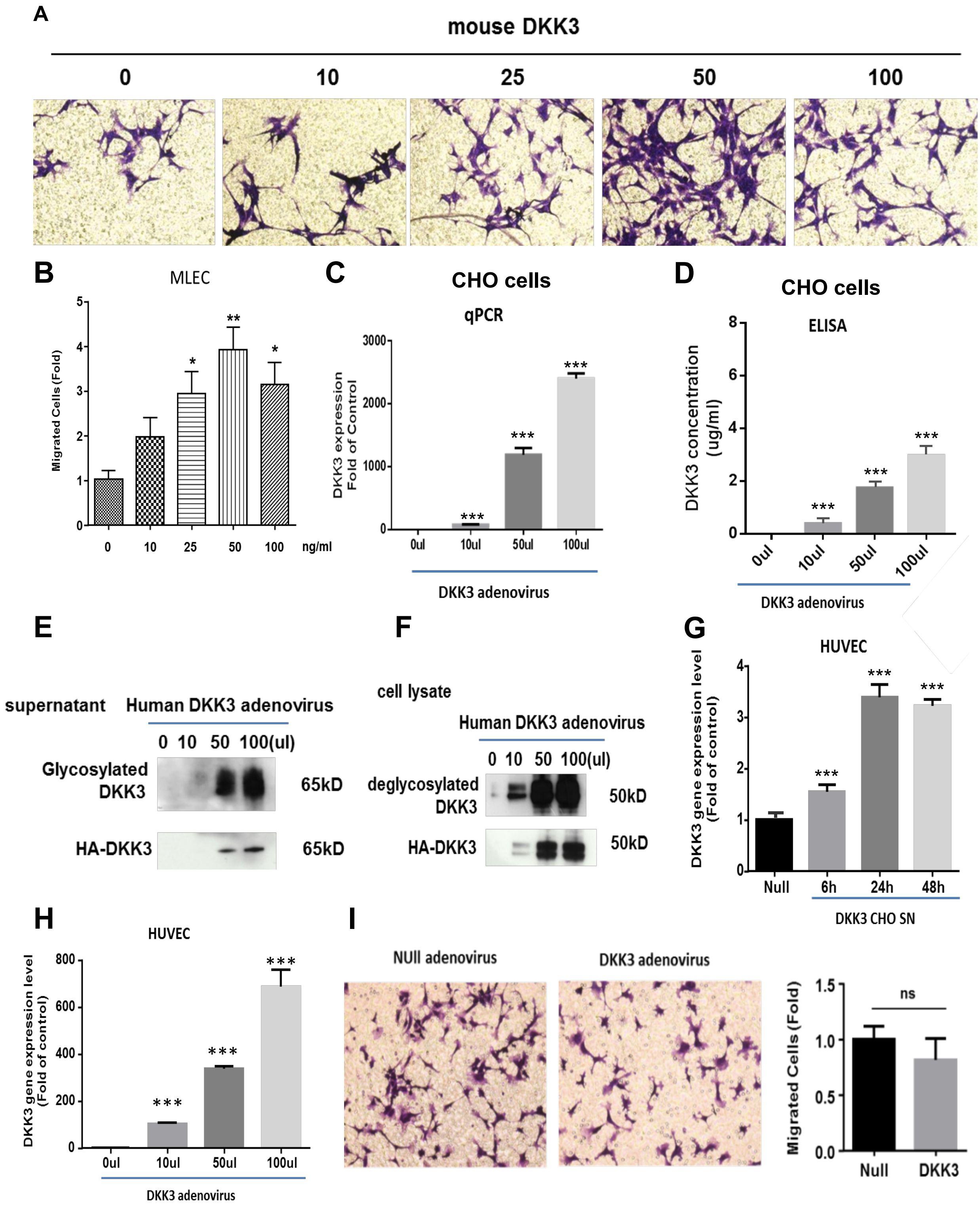

Figure S6. **Exogenous DKK3 induces endothelial cell migration.** **(A)** Transwell assay was performed on mouse lung endothelial cells (MLEC) that migrated toward serum free media containing indicated concentrations of murine recombinant DKK3. Scale bars, 100µm. **(B)** The quantification of MLEC migration is shown as mean ± SEM, n=3. *p<0.05. **p<0.01. qPCR **(C)** and ELISA **(D)** analysis were performed on Adeno-DKK3-transfected CHO cell lysate or supernatant, respectively, to analyze mRNA and protein levels of DKK3 after overexpression with human DKK3 adenovirus in CHO cell. Significant difference from the controls (n=3), ***p<0.001. Glycosylation or de-glycosylation of DKK3 were detected by both anti-human DKK3 antibody and anti-HA antibody in either supernatant **(E)** or CHO cell lysate **(F)** using Western blotting. **(G)** qPCR analyzed for DKK3 gene expression in HUVECs treated with Adeno-DKK3-transfected CHO supernatant at indicated time points. Significant difference from the controls (n=3), ***p<0.001. **(H)** Quantification of DKK3 gene expression in HUVECs after Adeno-DKK3 transfection for 48 hours. Significant difference from the controls (n=3), ***p<0.001. I. Transwell assay was performed to analyze the random migratory ability of HUVECs which transfected with either Null or DKK3 adenovirus. ns, not significance.

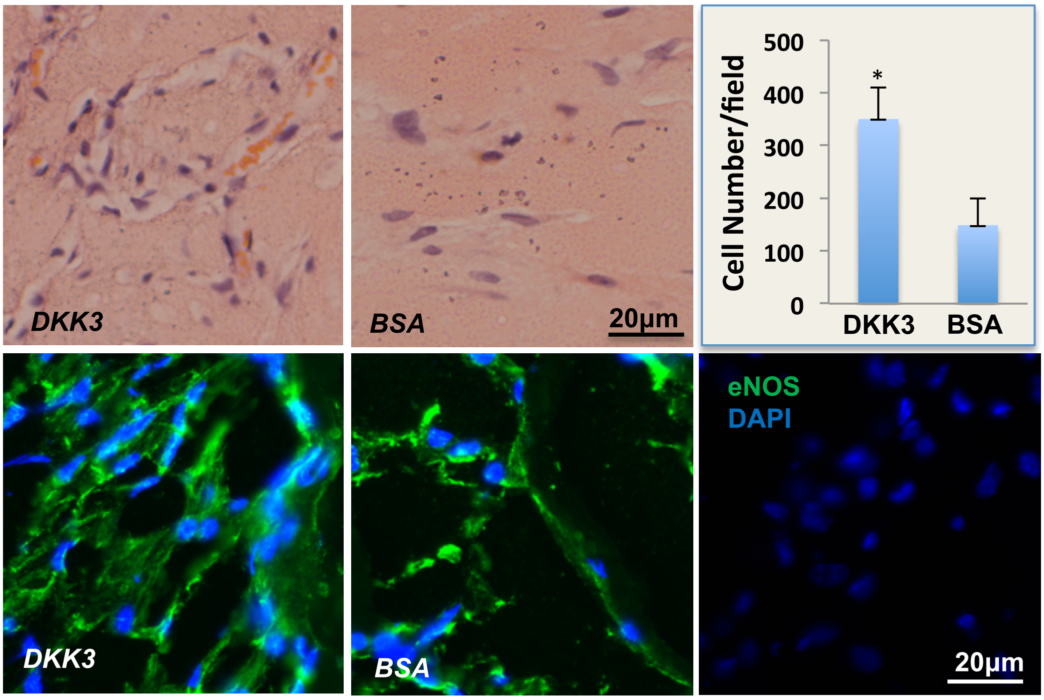

Figure S7. DKK3 enhanced endothelial migration in Matrigel plug assay. DKK3 or BSA (100mg/ml) was mixed with Matrigel and injected subcutaneously into mice. Two weeks after injection, Matrigel plugs were harvested and stained for HE (upper panel). Cell numbers within the plug were enumerated. Quantification data are means ± SEM of two groups (n=3), *p<0.05. Frozen sections were stained with EC specific marker eNOS demonstrating that DKK3 plug displayed well-formed endothelial structures and increased cell number compared to the BAS control.

Figure S8

Yu et al.

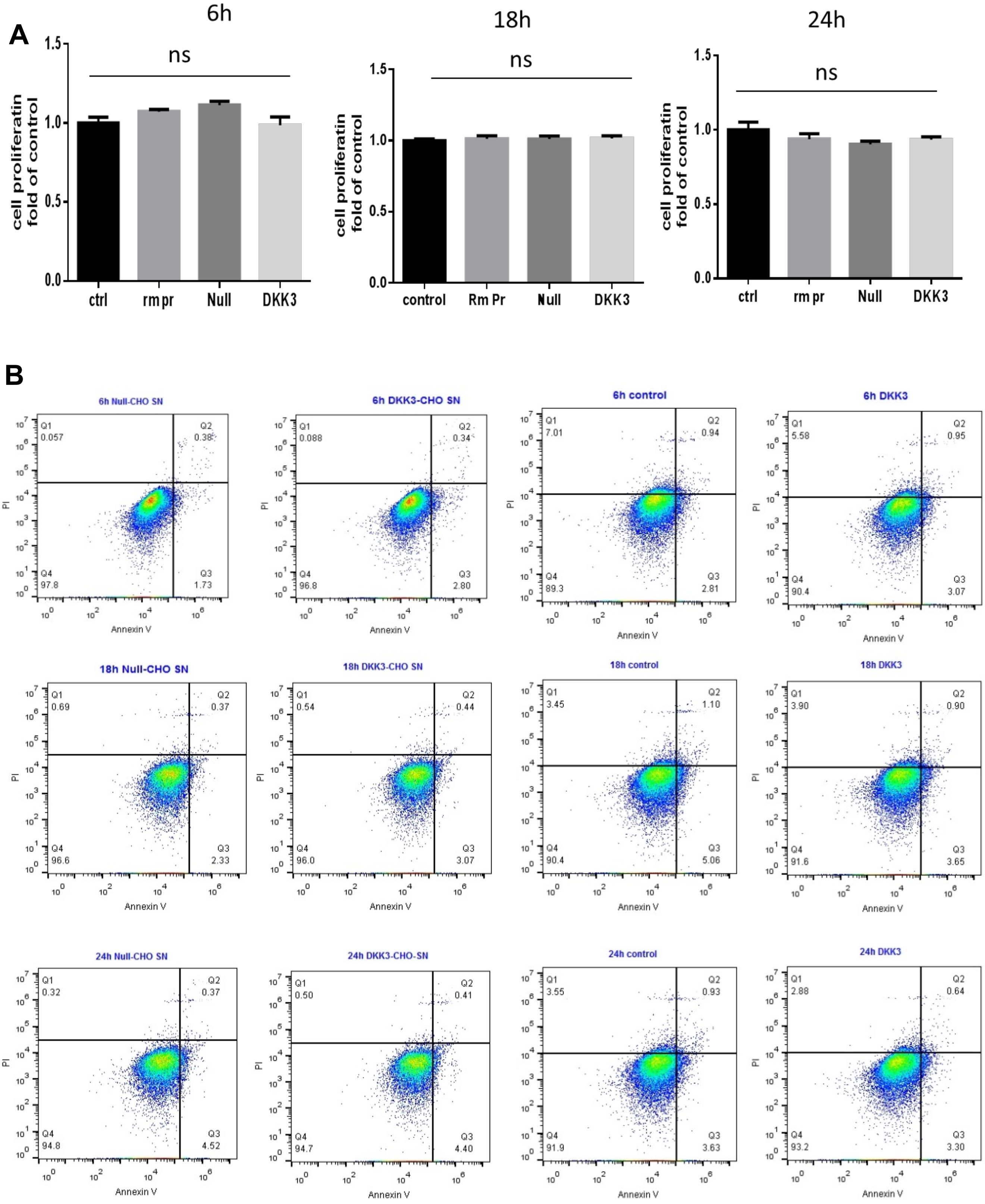

Figure S8. DKK3 has no effect on HUVEC proliferation and apoptosis. (A) Proliferation of HUVECs with treatments of either human recombinant DKK3 or Adeno-DKK3-transfected CHO supernatant at indicated times was evaluated by BrdU incorporation assay. ctrl, control, serum free medium, served as negative control of human recombinant DKK3; Rmpr, recombinant DKK3 protein; Null, Adeno-Null-transfected CHO supernatant, served as negative control of Adeno-DKK3-transfected CHO supernatant; DKK3, Adeno-DKK3-transfected CHO supernatant. **(B)** FACS analysis was also performed on HUVECs with incubation of either recombinant DKK3 or overexpressed-CHO supernatant at different time points followed by staining of Annexin V antibody or Propidium Iodide. In each dot plot, dots in Q3 (lower right) indicates early apoptotic cells; dots in Q2 (upper right) indicates necrotic cells, the number in each grid indicates cell percentage of the whole population.

Figure S9 **Yu et al.**

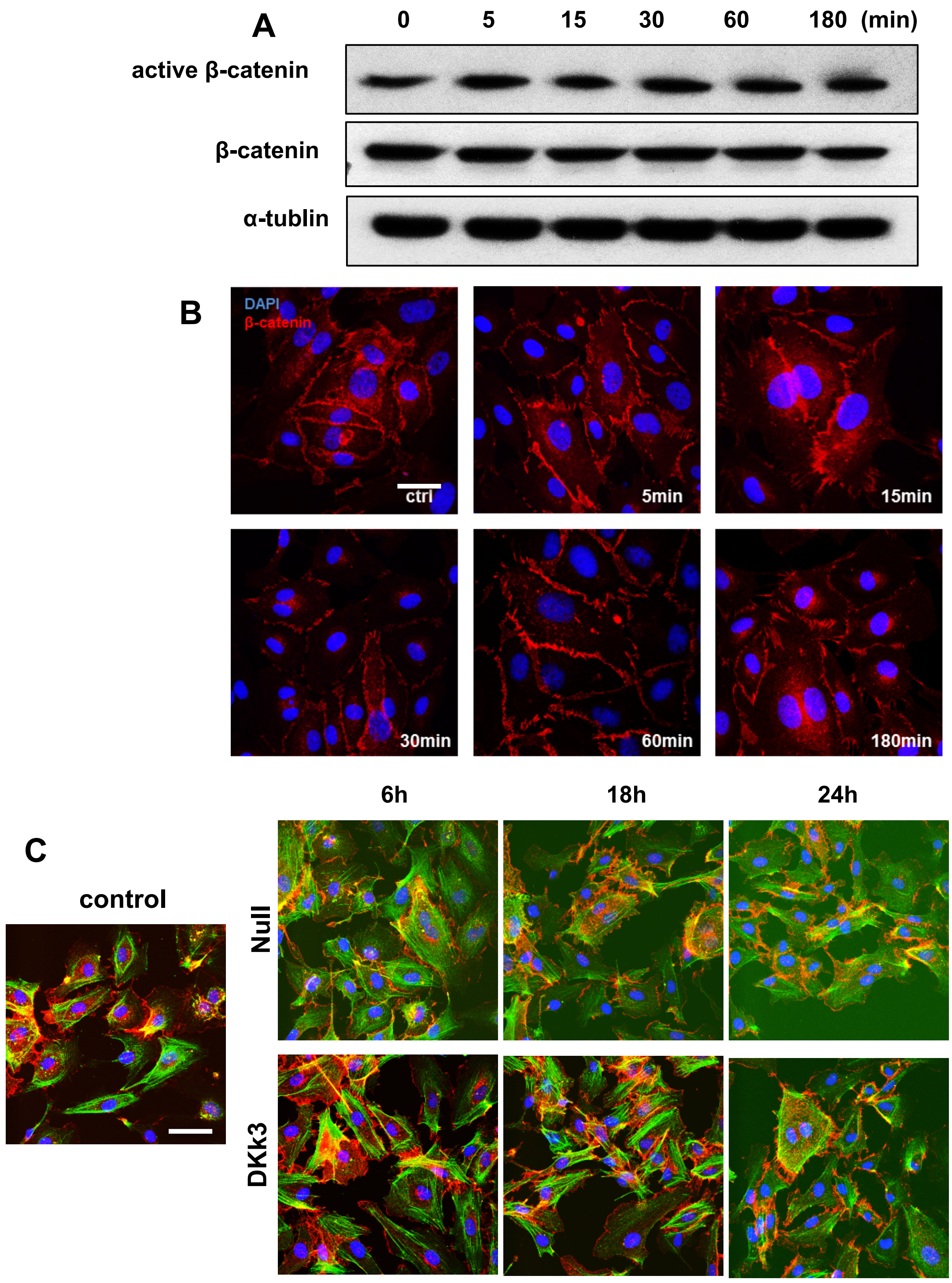

Figure S9. DKK3-induced HUVEC migration does not activate β-catenin. (A) Western blotting shows the expression of active and total β-catenin in HUVECs after treatment with human DKK3 at indicated time points. Immunofluorescence staining of DKK3 on HUVECs with either recombinant DKK3 **(B)** or Adeno-DKK3-transfected CHO supernatant **(C)** stimulation to identify the location of DKK3 expression.

A

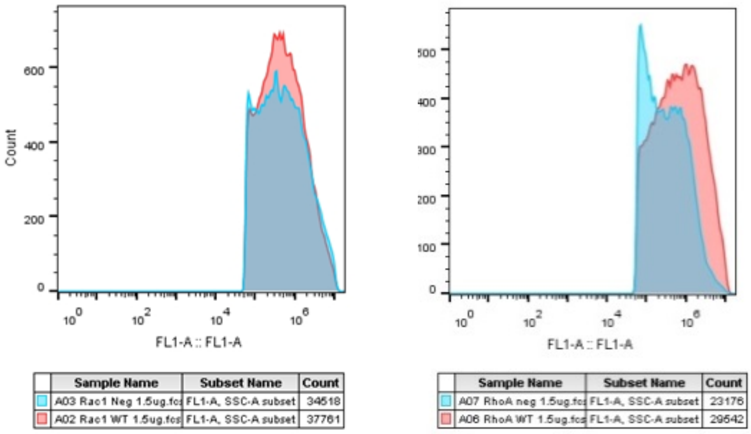

B

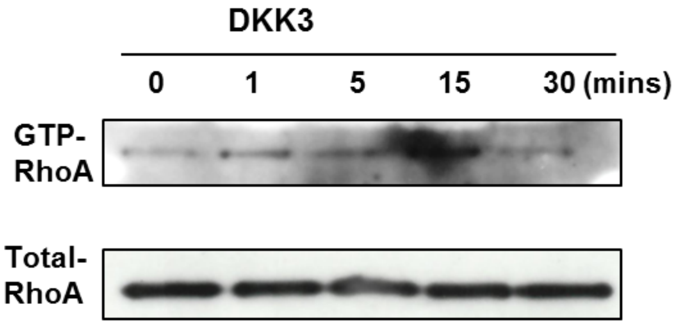

Figure S10. DKK3 has no effect on RhoA activation. (A) The histograms represent the eGFP positive HUVECs after transfection with eGFP-labeled plasmids. **(B)** Pull down assay was performed to analyze activated RhoA (GTP-RhoA) in HUVECs after stimulation with DKK3 at different time points.

Figure S11

Yu et al.

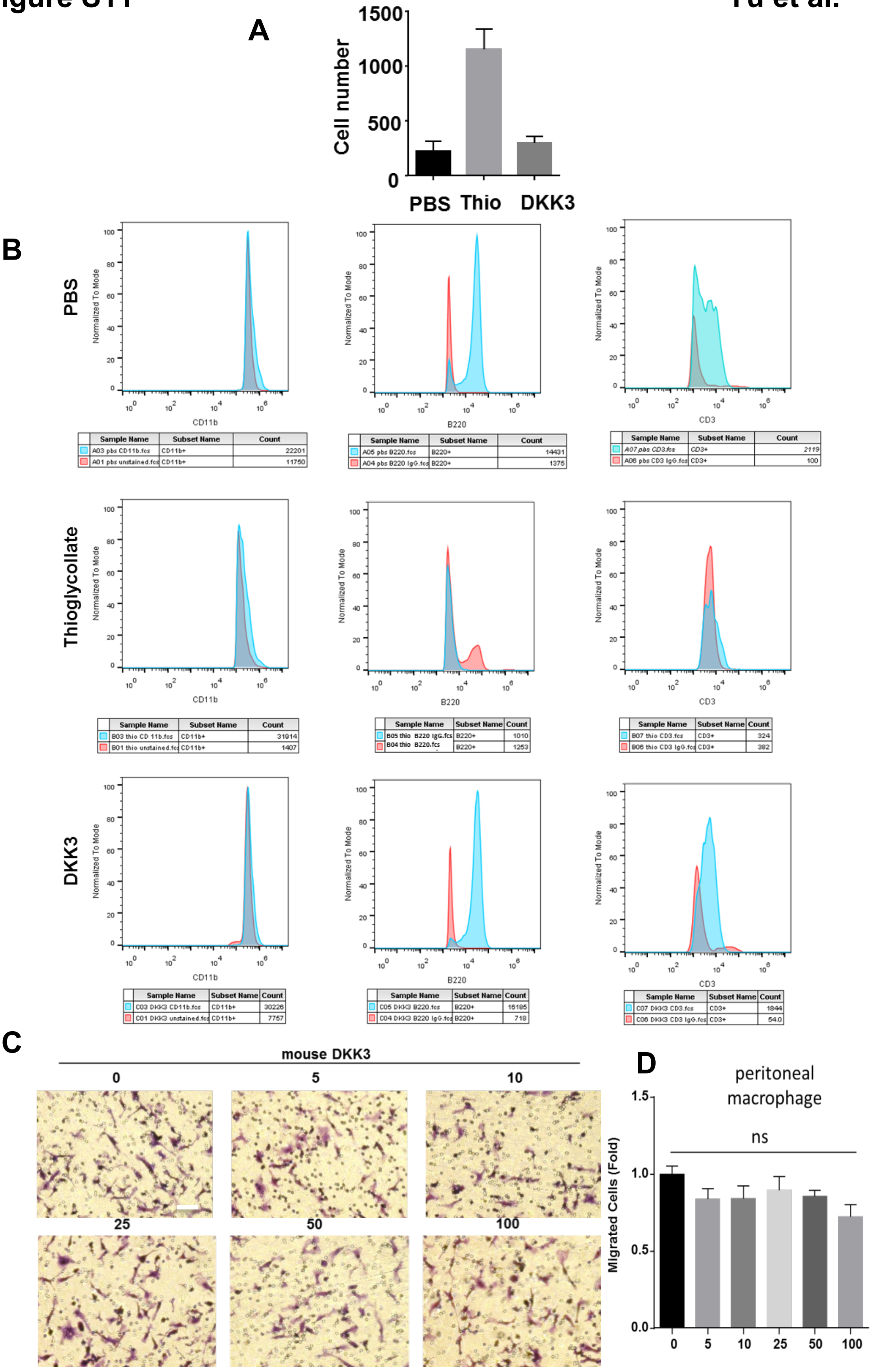

Figure S11. DKK3 does not show a role in leukocytes recruitment and macrophage migration. (A) The peritoneal cells isolated from mice that 3 days after injection with saline, thioglycollate or mouse recombinant DKK3 were counted under the microscope and the graph shows the cell number per milliliter of each group. **(B)** Flow cytometry analysis shows the cell components in the whole cell population of each group. **(C)** Migration towards mouse recombinant DKK3 was evaluated on the peritoneal macrophages which isolated from thiglycollate stimulation in vivo by using Transwell assay. **(D)** The quantification of peritoneal macrophages migration was shown in mean ± SEM. ns, no significance.

Figure S12 Yu et al.

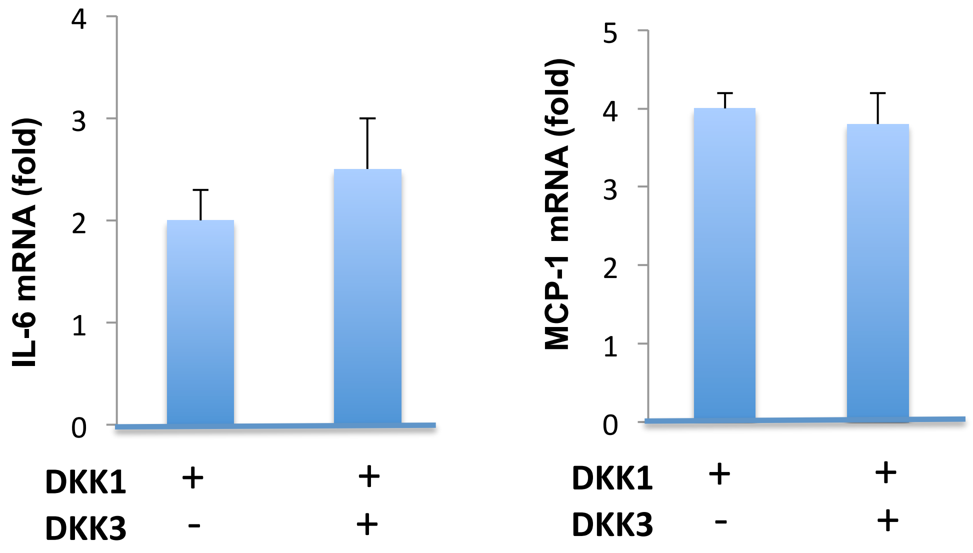

Figure S12. DKK3 pretreatment did not influence DKK1-induced cytokine expression. HUVECs were pre-incubated with DKK3 (50 ng/ml) for 1 hour and then DKK1 (50ng/ml) was added and incubated for 12 hours. The cells were harvested and qPCR for IL-6 and MCP-1 were performed. Data are means±SEM of three experiments. Note there is no significant difference between two groups.

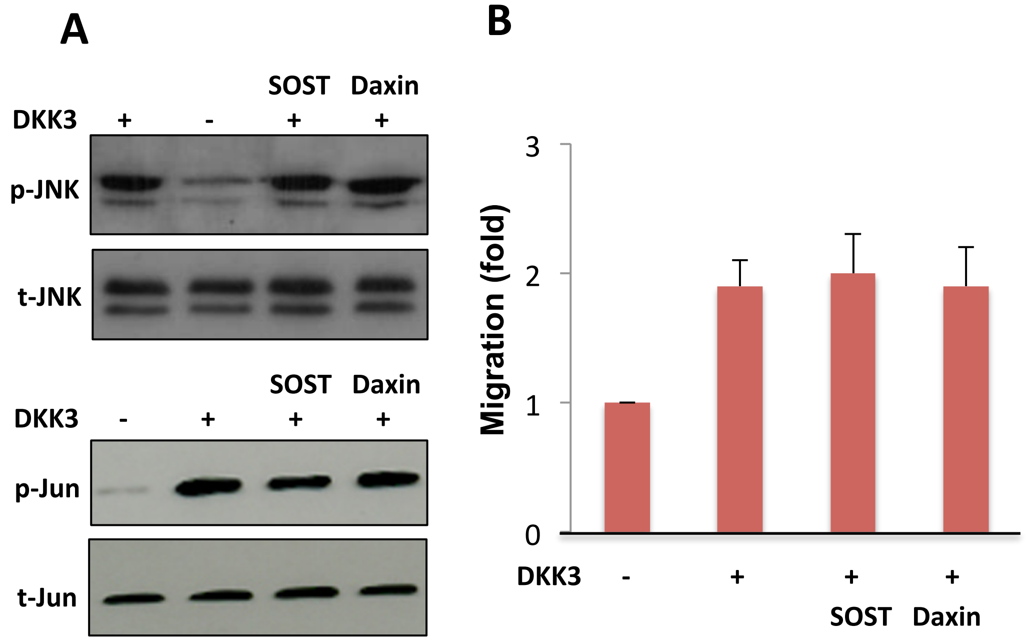

Figure S13. LRP5 and LRP6 antagonists did not block DKK3-induced signalling and cell migration. Panel A, HUVECs were pre-incubated with SOST/Sclerostin or Draxin (50 ng/ml) for 1 hour and then DKK3 (25 ng/ml) was added and incubated for 30 minutes. The cells were harvested and Western blots for JNK and Jun were performed. Panel B, HUVECs were pre-incubated with SOST/Sclerostin or Draxin (50 ng/ml) for 1 hour and then cell migration assay against DKK3 (25 ng/ml) was performed. Note that LRP5 and LRP6 antagonists SOST/ Sclerostin or Draxin did not block the activity of DKK3 in endothelial migration.

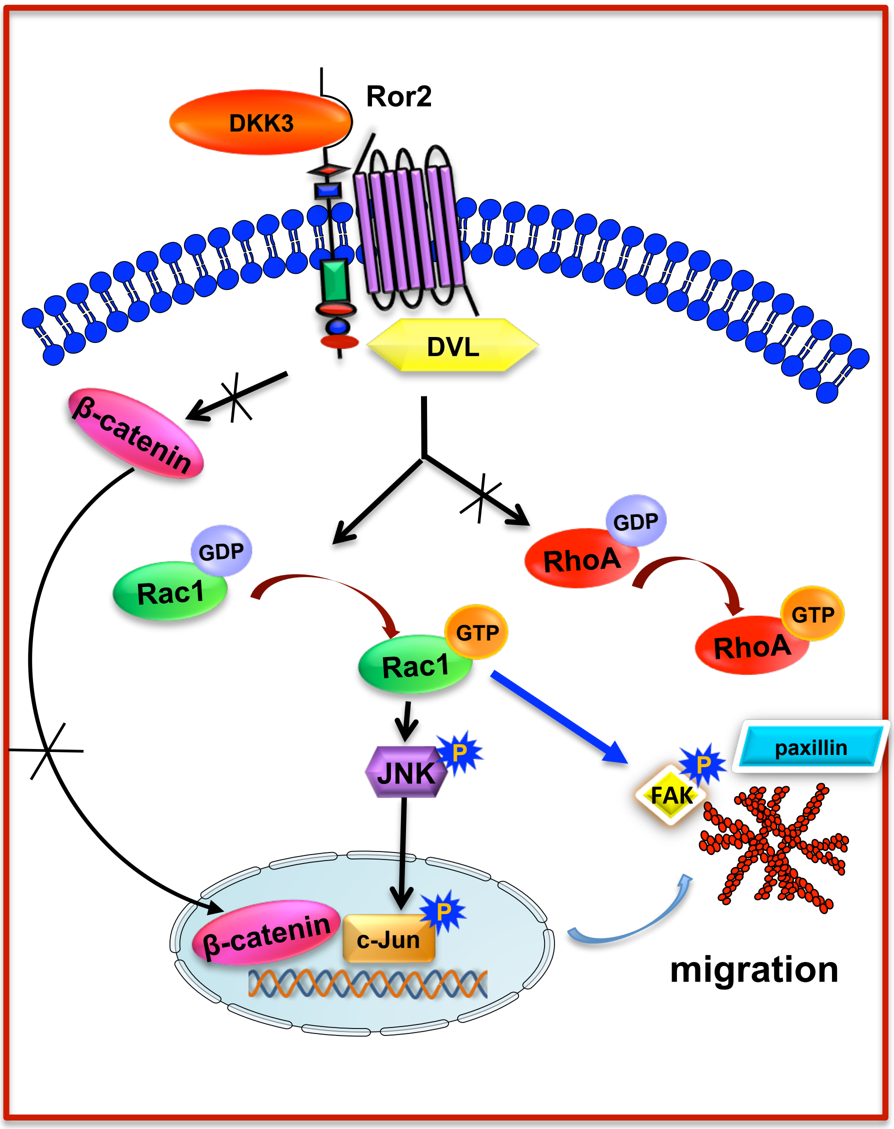

Figure S14. Schematic representation of the mechanism by which DKK3 induced endothelial cell migration. Soluble DKK3 can binds to Ror2/DVL on the surface of endothelial cells leading to Rac1 activation, which in turn activates JNK. JNK phosphorylates c-Jun that binds to the promoter region of genes, leading to cytoskeleton rearrangement. The overall effect favorites endothelial cell migration.
